# Supplementary material for: Anaesthesia Choice for Creation of Arteriovenous Fistula (ACCess) study protocol : a randomised controlled trial comparing primary unassisted patency at 1 year of primary arteriovenous fistulae created under regional compared to local anaesthesia
Source: BMJ Open. 2021 Dec 22;11(12):e052188. doi: 10.1136/bmjopen-2021-052188 (PMC8704953; doi:10.1136/bmjopen-2021-052188)
Supplement: Supplementary data [file bmjopen-2021-052188supp001.pdf]

Appendix 1. Preparation of anaesthetic mixture for RA and administration of brachial plexus block.

Preparation:
Prepare 2 x 20ml syringes.
To each syringe add:

- 10ml 0.5% Levobupivacaine
- 10ml 1% Lidocaine
- 0.05ml 1:1,000 Adrenaline

Administration of brachial plexus block:
A minimum volume based on weight (outlined below) must be injected during the initial block (whether supraclavicular or axillary).

- 45-50kg: 15ml
- 51-60kg: 20ml
- >60kg: 25ml

Larger volumes may be used at the discretion of the anaesthetist as long as maximum dose limits are observed, remembering that local anaesthetic may also be required for surgical supplementation and that these doses are additive. Consider using ideal body weight in obese patients.
