# Supplementary material for: Anaesthesia Choice for Creation of Arteriovenous Fistula (ACCess) study protocol : a randomised controlled trial comparing primary unassisted patency at 1 year of primary arteriovenous fistulae created under regional compared to local anaesthesia
Source: BMJ Open. 2021 Dec 22;11(12):e052188. doi: 10.1136/bmjopen-2021-052188 (PMC8704953; doi:10.1136/bmjopen-2021-052188)
Supplement: Supplementary data [file bmjopen-2021-052188supp002.pdf]

ACCESS

Anaesthesia Choice for Creation of ArtEriovenous FiStulae (ACCess study)

Chief Investigator: Emma Aitken

Participant Identification Number:

Please initial each box if you agree with the following statements:

I confirm that I have read and understood the information sheet dated *XX/XX/XX* (version *X.X)* for the above study. I have had the opportunity to consider the information, ask questions and have had those questions answered satisfactorily.	
I understand that my participation is voluntary and that I am free to withdraw at any time without giving any reason, without my medical care or legal rights being affected.	
I understand that relevant sections of my medical notes and data collected during the study may be looked at by individuals from the research team, regulatory authorities or from the Sponsor, NHS Greater Glasgow and Clyde, where it is relevant to my taking part in the research. I give permission to these individuals to have access to my records. I understand that my data will be held by the University of Glasgow.	
I understand that the information collected about me will be used to support other research in the future, and may be shared anonymously with other researchers.	
I agree to the study team having my phone number for the purpose of contacting me during the study.	
I agree to take part in the above study.	

**Name of Participant**	**Signature**	**Date**
....	....	....
**Name of Researcher**	**Signature**	**Date**
....	....	....

When completed: 1 for participant; 1 for researcher site file; 1 to be kept in medical notes.

IRAS ID: 290482
