# Supplementary material for: Anaesthesia Choice for Creation of Arteriovenous Fistula (ACCess) study protocol : a randomised controlled trial comparing primary unassisted patency at 1 year of primary arteriovenous fistulae created under regional compared to local anaesthesia
Source: BMJ Open. 2021 Dec 22;11(12):e052188. doi: 10.1136/bmjopen-2021-052188 (PMC8704953; doi:10.1136/bmjopen-2021-052188)
Supplement: Author's manuscript [file bmjopen-2021-052188.draft_revisions.pdf]

BMJ Open

BMJ Open is committed to open peer review. As part of this commitment we make the peer review history of every article we publish publicly available.

When an article is published we post the peer reviewers' comments and the authors' responses online. We also post the versions of the paper that were used during peer review. These are the versions that the peer review comments apply to.

The versions of the paper that follow are the versions that were submitted during the peer review process. They are not the versions of record or the final published versions. They should not be cited or distributed as the published version of this manuscript.

BMJ Open is an open access journal and the full, final, typeset and author-corrected version of record of the manuscript is available on our site with no access controls, subscription charges or pay-per-view fees (http://bmjopen.bmj.com).

If you have any questions on BMJ Open's open peer review process please email info.bmjopen@bmj.com

BMJ Open

The Anaesthesia Choice for Creation of arteriovEnouS fiStula (ACCess) study : a multicentre, observer-blinded, randomised controlled trial comparing primary unassisted patency at one year of primary radio-/brachio-cephalic arteriovenous fistulae created under regional compared to local anaesthesia.

Journal:	*BMJ Open*
Manuscript ID	bmjopen-2021-052188
Article Type:	Protocol
Date Submitted by the Author:	10-Apr-2021

Complete List of Authors:	Macfarlane, Alan; NHS Greater Glasgow and Clyde, Anaesthesia; University of Glasgow,<br>Kearns, Rachel J; NHS Greater Glasgow and Clyde, Anaesthesia; University of Glasgow, Anaesthesia<br>Clancy , Marc James; Queen Elizabeth University Hospital Campus, Renal Surgery; University of Glasgow, Institute of Cardiovascular and Medical Sciences<br>Kingsmore, David; Queen Elizabeth University Hospital, Department of Vascular Surgery; University of Glasgow, Institute of Cardiovascular and Medical Sciences<br>Stevenson, Karen; Queen Elizabeth University Hospital, Department of Renal Surgery<br>Jackson, Andrew; Queen Elizabeth University Hospital, Department of Renal Surgery<br>Mark, Patrick; University of Glasgow, Institute of Cardiovascular & Medical Sciences<br>Aitken, Margaret; Queen Elizabeth University Hospital, Department of Renal Surgery<br>Moonsinghe, Ramani; University College London, Centre for Perioperative Medicine<br>Vindrola-Padros, Cecilia; University College London, Department of Targeted Intervention<br>Gaianu, Lucian; Independent Health Economist, Healthonomics UK Ltd<br>Pettigrew, Gavin ; University of Cambridge, Surgery<br>Motallebzadeh, Reza; Royal Free London NHS Foundation Trust, Department of Nephrology and Transplantation; University College London, Department of Surgery and Interventional Science<br>Karydis, Nikolaos; Guy's and St Thomas' NHS Foundation Trust, Department of Transplantation<br>Vesey, Alex; University Hospital Hairmyres, Department of Vascular Surgery<br>Singh, Rita; Freeman Hospital, Department of Anaesthesia<br>Muniraju, Thalakunte; Dumfries and Galloway Acute Hospitals, Department of Nephrology<br>Suttie, Stuart; Ninewells Hospital and Medical School, Department of

	Vascular Surgery<br>McConnachie, Alex; University of Glasgow, Robertson Centre for Biostatistics<br>Wetherall, Kirsty; University of Glasgow, Robertson Centre for Biostatistics<br>El-Boghdadly, Kariem; Guy's and Saint Thomas' NHS Foundation Trust, Department of Anaesthesia and Perioperative Medicine; King's College London<br>Hogg, Rosemary; Belfast Health and Social Care Trust, Department of Anaesthesia<br>Thomson, Iain; Queen Elizabeth University Hospital, Department of Anaesthesia<br>Nangalia, Vishal; Royal Free London NHS Foundation Trust, Department of Anaesthesia<br>Aitken, Emma; Queen Elizabeth University Hospital, Department of Renal Surgery

Keywords:	Adult anaesthesia < ANAESTHETICS, End stage renal failure < NEPHROLOGY, Vascular surgery < SURGERY, Transplant surgery < SURGERY

SCHOLARONE™
Manuscripts

I, the Submitting Author has the right to grant and does grant on behalf of all authors of the Work (as defined in the below author licence), an exclusive licence and/or a non-exclusive licence for contributions from authors who are: i) UK Crown employees; ii) where BMJ has agreed a CC-BY licence shall apply, and/or iii) in accordance with the terms applicable for US Federal Government officers or employees acting as part of their official duties; on a worldwide, perpetual, irrevocable, royalty-free basis to BMJ Publishing Group Ltd ("BMJ") its licensees and where the relevant Journal is co-owned by BMJ to the co-owners of the Journal, to publish the Work in this journal and any other BMJ products and to exploit all rights, as set out in our licence.

The Submitting Author accepts and understands that any supply made under these terms is made by BMJ to the Submitting Author unless you are acting as an employee on behalf of your employer or a postgraduate student of an affiliated institution which is paying any applicable article publishing charge ("APC") for Open Access articles. Where the Submitting Author wishes to make the Work available on an Open Access basis (and intends to pay the relevant APC), the terms of reuse of such Open Access shall be governed by a Creative Commons licence – details of these licences and which Creative Commons licence will apply to this Work are set out in our licence referred to above.

Other than as permitted in any relevant BMJ Author's Self Archiving Policies, I confirm this Work has not been accepted for publication elsewhere, is not being considered for publication elsewhere and does not duplicate material already published. I confirm all authors consent to publication of this Work and authorise the granting of this licence.

For peer review only

Title :

The Anaesthesia Choice for Creation of arteriovEnouS fiStula (ACCess) study : a multicentre, observer-blinded, randomised controlled trial comparing primary unassisted patency at one year of primary radio-/brachio-cephalic arteriovenous fistulae created under regional compared to local anaesthesia.

Authors :

Alan JR Macfarlane[1,2], Rachel J Kearns[1,2], Marc Clancy[3,4], David Kingsmore[4,5], Karen Stevenson[3], Andrew Jackson[3], Patrick B Mark[4], Margaret Aitken[3], Ramani Moonesinghe[6,7,8], Cecilia Vindrola-Padros[7], Lucian Gaianu[9], Gavin Pettigrew[10], Reza Motallebzadeh[8,11], Nikolaos Karydis[12], Alex Vesey[13], Rita Singh[14], Thalakunte Muniraju[15], Stuart Suttie[16], Alex McConnachie[17], Kirsty Wetherall[17], Kariem El Boghdadly[18,19], Rosemary Hogg[20], Iain Thomson[21], Vishal Nangalia[22], Emma Aitken[3,4]. On behalf of the ACCess collaborative group.

Corresponding author :

Professor Alan Macfarlane

Consultant Anaesthetist

Glasgow Royal Infirmary, 84 Castle Street, Glasgow, G4 0SF, UK

Honorary Professor, University of Glasgow, Glasgow, UK

Telephone: +44 (0)141 201 3870

Email: alan.macfarlane@ggc.scot.nhs.uk

Word Count : 3971

For peer review only

Abstract

Introduction

Arteriovenous fistulae (AVF) are the “gold standard” vascular access for haemodialysis. Universal usage is limited however by a high early failure rate. Several small, single-centre studies have demonstrated better early patency rates for AVF created under regional anaesthesia compared to local anaesthesia. The mechanistic hypothesis is that the sympathetic blockade associated with regional anaesthesia causes vasodilatation and increased blood flow through the new AVF. Despite this, considerable variation in practice exists in the UK. A high quality, adequately powered, multicentre randomised controlled trial (RCT) is required to definitively inform practice.

Methods and analysis

The Anaesthesia Choice for Creation of arteriovEnouS fiStula (ACCess) study is a multicentre, observer-blinded, RCT comparing primary radio-/brachio-cephalic AVF created under regional versus local anaesthesia. The primary outcome is primary unassisted AVF patency at one year. Access-specific (e.g. stenosis/ thrombosis), patient-specific (including health-related quality of life (HRQoL)) and safety secondary outcomes will be evaluated. Health economic analysis will also be undertaken.

Ethics and dissemination

The ACCess study has been approved by the West of Scotland Research and Ethics Committee Number 3 (20/WS/0178). Results will be published in open access peer-reviewed journals within 12 months of completion of the trial. We will also present our

For peer review only

findings at key national and international renal and anaesthetic meetings, and support dissemination of trial outcomes via renal patient groups.

Registration details

Trial registration number : ISRCTN14153938 (registered: 11th November 2020)

Sponsor: NHS Greater Glasgow and Clyde GN19RE456; Protocol Version 1.1 (29th January 2021)

REC/IRAS ID: 290482

Strengths and limitations of this study

- This is a prospective, multicentre randomised, observer blinded trial designed to examine whether primary radio-/brachio-cephalic arteriovenous fistulae created under regional anaesthesia rather than local anaesthesia have better one-year primary unassisted functional patency.
- With 566 participants, this will be the largest trial to date comparing regional to local anaesthesia and will address criticisms of previous smaller, single centre randomised trials.
- An associated cost-effectiveness analysis will provide sufficient evidence to guide practice and policy.
- The main limitation of this study is that in pre-dialysis patients, the primary endpoint uses surrogate markers of fistula patency (clinical assessment and ultrasound (USS) criteria) rather than successful use of the fistula for haemodialysis.

For peer review only

Introduction

The incidence of kidney failure requiring kidney replacement therapy (KRT) has increased substantially over the last 30 years and over 25,000 people in the UK are currently treated with maintenance haemodialysis (HD).[1] Kidney disease has a significant impact on both longevity and quality of life and places considerable demand on healthcare resources.[2] Vascular access is “a major modifiable risk factor” in terms of patient experience and outcome on HD, with arteriovenous fistulae (AVF) being the preferred mode of vascular access.[3] Patients dialysing via AVF experience less infective and thrombotic complications, and are three times less likely to be admitted to hospital than their counterparts with central venous catheters (CVCs).[3] Such frequent hospitalisations have a negative impact on health-related quality of life (HRQoL).[4] Furthermore, both quality of dialysis and patient survival are superior when comparing dialysis via AVF with central venous catheters (CVCs) or prosthetic arteriovenous grafts (AVGs).[5]

Despite these benefits, universal adoption of AVF remains suboptimal. The most recent UK Renal Registry (UKRR) Multisite Dialysis Access Audit highlighted that nearly 80% of dialysis units in England, Wales and Northern Ireland still fail to achieve Renal Association targets which recommend 60% of incident patients receive HD via AVF or AVG.[6,7] One principal obstacle to the widespread utilisation of AVF is “failure to mature”, with early failure rates approaching 50%.[8,9] Any intervention that improved AVF maturation would confer significant benefit to patient health and wellbeing, reduce surgical workload and deliver cost savings. Anaesthetic technique is one factor believed to influence AVF maturation and outcome.

For peer review only

Regional anaesthesia (RA), unlike local anaesthesia (LA), generates sympathetic blockade which results in vasodilatation, improved tissue oxygenation and increased blood flow through the new AVF.[8,10] Several studies have demonstrated superior short-term patency rates of AVF created under brachial plexus block (BPB) compared to LA.[8,11] In the only RCT to date with prolonged follow-up, RA improved both immediate and one-year functional AVF patency compared to LA.[12] A concomitant health economic analysis using HRQoL data extrapolated from the literature established net cost savings at one-year and an incremental cost-effectiveness ratio of approximately £12,900 per quality-adjusted life year (QALY) gained over a 5-year time horizon with RA.

Both European Society for Vascular Surgery and European Renal Association guidelines suggest considering using RA for all primary AVF.[13,14] The Kidney Disease Outcomes Quality Initiative Vascular Access guidelines disagree, stating choice of anaesthesia should be based on operator discretion.[15] Such disparity regarding the choice of anaesthesia for AVF creation is reflected across UK centres, with significant variation in practice.[16] Whilst this is in part due to a lack of anaesthetic availability or capability, the failure to modify local practices perhaps also reflects the lack of strong evidence. All RCTs to date have been single-centre, with some suffering from methodological flaws.[11] Further, more robust, health economics analysis is required to establish whether any potential durable clinical benefit could be offset against the longer procedural times, need for a skilled anaesthetist and the additional upfront costs associated with RA. Only a definitive, adequately powered, multicentre RCT with associated cost-effectiveness analysis will provide sufficient evidence to change practice and policy.

For peer review only

Methods and Analysis

The ACCess study is a multicentre, observer-blinded, parallel group, superiority RCT with an internal pilot and embedded process evaluation study. The primary objective is to compare unassisted functional AVF patency at one year in patients undergoing primary radio- (RCF) or brachio-cephalic fistula (BCF) creation under RA versus LA. The participant timeline is outlined in Figure 1.

Participants

Patients will be recruited from high (>150 cases per year) and medium (>50 cases per year) volume UK centres providing vascular access for HD.

Recruitment

Potentially eligible participants will be identified from vascular access and/or 'pre-dialysis' (low clearance) clinics and theatre waiting lists by the clinical team. A vein mapping ultrasound will be performed to ensure minimum vessel characteristics. We anticipate 12-20 centres recruiting an average of 2 patients/centre/month. Recruitment is due to commence May 2021 and is anticipated to take two-years. Preliminary results are expected late 2024.

Inclusion criteria

For peer review only

All adult patients (older than 18 years old) with kidney failure requiring replacement therapy or chronic kidney disease (CKD) stage IV or V referred for primary RCF or BCF creation will be eligible.

Exclusion criteria

Exclusion criteria are outlined in Box 1.

Allocation

A central randomisation facility (interactive web response system, IWRS) at the Robertson Centre for Biostatistics (RCB), University of Glasgow, will randomise patients 1:1 to the intervention group (RA) or comparator group (LA). The randomisation list will be created by a computer-generated program, using a method of permuted blocks stratified by centre, dialysis status (pre-dialysis/HD) and site of AVF (RCF/BCF). Randomisation will take place at a patient, not centre, level to minimise bias from variation in surgical and dialysis practice. The randomisation list, the program that generated it and the random seed used will be stored in a secure network location, accessible only to those responsible for provision of the randomisation system. Clinicians responsible for delivering the perioperative care will perform the web-based randomisation.

Intervention

The choice of anaesthetic agents is influenced largely by the successful utilisation of these combinations in a previous study[8]; the ready availability of these drugs within the UK; acceptability to collaborating centres and the ability of the combination to provide both rapid onset and prolonged duration of block.[17]

For peer review only

Interventional arm: regional anaesthesia (RA)

An ultrasound guided supraclavicular or axillary BPB will be administered by a consultant anaesthetist trained in RA, or trainee practicing under direct supervision. The supraclavicular approach will be considered first-line, unless the anatomy or patient risk profile is unfavourable. In patients on antiplatelets or other anticoagulants, the choice between supraclavicular and axillary block will be at the anaesthetist's discretion, taking into account "compressibility, vascularity and consequences of bleeding".[18] Where pulmonary disease is present, an axillary nerve block eliminates the risk of pneumothorax or temporary phrenic nerve paralysis.

A 1:1 mixture of 0.5% L-bupivacaine and 1% lidocaine, mixed with epinephrine to 1 in 400,000 final concentration, will be utilised (Appendix 1). Maximum dose limits are 2 mg/kg for bupivacaine and 7 mg/kg for lidocaine with epinephrine, recognising the effects are additive. The volume of LA injected must account for patient weight and maximum dose limits, whilst considering the need for LA supplementation. In a study where the median patient weight was 66kg the ED_{95} for supraclavicular blocks was 27ml.[19] A minimum volume of 25ml must be injected for patients over 60kg, and reduced accordingly for lower patient weights (Appendix 1). A suggested supraclavicular technique involves depositing a minimum of 25% of LA in the "corner pocket" between the 1st rib and the subclavian artery and the remainder posterolateral to the plexus, avoiding deliberate intracluster injection.[20] For axillary blocks, the same minimum volumes must be utilised, targeting 25% of the LA to the musculocutaneous nerve, with the remainder deposited around the ulnar, median and radial nerves as well as the cutaneous nerves of the arm and forearm if visualised.

For peer review only

Sensory and motor block of musculocutaneous, median, radial and ulnar nerves will be recorded every 5 minutes using a validated 3-point scale.[21] Sensory blockade of the medial cutaneous nerve of the forearm and arm will also be recorded. Measurements will be continued until either sensory block is adequate or 30-minutes has elapsed, at which point the block may be supplemented by targeted ultrasound-guided axillary or midhumeral injection as appropriate.

Comparator Arm: Local Anaesthesia (LA)

A 1:1 mixture of 0.5% L-bupivacaine and 1% lidocaine will be infiltrated around the operative site by the operating surgeon. After 5 minutes, adequacy of anaesthesia will be tested by application of a painful stimulus and additional LA infiltration administered as required. Maximum dose limits of 2 mg/kg for bupivacaine and 3mg/kg for lidocaine will be observed, recognising the effects are additive.

Management of a "failed block" (or failure of local anaesthesia)

A "failed block" will be defined as any block that despite the targeted intervention described above requires additional supplementation with LA, analgesia, conversion to GA or abandonment of surgery. The algorithm for "failed block" or "failed LA" will be as follows:

1. Supplementation at surgical site with LA (1% lidocaine) up to maximum cumulative LA dosage
2. Intravenous sedation and analgesia at the discretion of the anaesthetist
3. General anaesthesia
4. Abandonment of procedure: decision to be made following discussion between operating surgeon and anaesthetist if deemed unsafe to proceed with GA

For peer review only

The Trial Steering Committee (TSC)/Independent Data Monitoring Committee (IDMC) will monitor the number of failed blocks for patient safety and quality assurance throughout the study.

Fistula surgery

A standard approach to the vessels will be via a transverse incision at, or just below, the elbow crease for BCF and longitudinal or curvilinear incision at the wrist for RCF. The cephalic vein (or median cubital vein if suitable) will be dissected and skeletalised for a short length proximally and distally. Visible branches will ligated and divided. The vein will be divided, spatulated where appropriate and flushed with heparinised saline. The artery will be dissected and controlled with bulldog clamps or slings. The decision to utilise median cubital, perforating branch or true outflow cephalic vein for the anastomosis will be at the surgeon's discretion, as will be the decision to create a proximal radial or ulnar-cephalic fistula at the elbow. The size of the arteriotomy will be based on individual patient risks and benefits but arteriotomies will generally be between 3-5mm in length on the brachial artery and 7-10mm on the radial artery. An end-to-side anastomosis of vein to artery will be performed with continuous 6.0 (elbow) or 7.0 (wrist) Prolene.

Blinding

Due to the systemic effects of RA (motor blockade; visible venodilatation etc.), which do not occur with LA, it will not be possible to blind the patients, surgical or anaesthetic teams. Dialysis staff, and staff performing follow-up visits will be blinded to the intervention. USS will also provide independent objective assessment of the AVF. The statisticians and health economist will be blinded to the intervention.

For peer review only

Outcomes

The primary outcome is unassisted functional AVF patency at one year, defined as the ability of the AVF to uninterruptedly deliver the prescribed dialysis without intervention.[22] In pre-dialysis patients, this will be assessed both clinically by an experienced, blinded dialysis nurse and ultrasonographically, the target being 4mm diameter and access flow >500ml/min.[23]

All secondary outcome measures will be assessed at 3 and 12 months. These were chosen with two considerations: patient-centred care and to facilitate health economic analysis. The secondary outcomes reflect the "standard CKD set" recommended by the International Consortium for Health Outcomes Measurement (ICHOM) CKD Working Group.[24] Key safety and efficacy outcomes for USS-guided regional nerve blocks outlined by the National Institute for Health and Clinical Excellence (NICE) will be recorded.[25] Secondary outcome measures are listed in Box 2.

Economic evaluation

A cost-effectiveness analysis will be conducted alongside the clinical trial. The health outcomes for the cost-effectiveness analysis will be QALYs. Two complementary cost-effectiveness analyses will be performed, namely a within-trial evaluation where cost and health effects of individual patients are limited to the one-year follow-up period in the trial and a decision model approach where effects are modelled to incorporate longer-term impacts of the intervention.

For peer review only

The primary outcome of the economic evaluation is the incremental cost-effectiveness ratio of RA compared to LA in AVF creation expressed in £/QALY. All intervention resource use and access-related resource use will be available from the secondary outcome data and unit costs will be applied to all resource use estimates, informed where possible from standard UK sources. A bottom-up approach will be used to estimate the costs associated with the two anaesthesia procedures. Effects will be captured at the individual patient level and QALYs will be derived by combining overall survival with utility weights derived from the EQ-5D questionnaire values obtained at the pre-operative time, at 3 months and 12 months after treatment.

At the end of the trial, cost and effects will be calculated for each patient, with the effect being individual QALYs derived by approximating the area under the curve of the EQ-5D index values obtained during the one-year follow- up. A point estimate of the incremental cost-effectiveness ratio (ICER) at one year will be calculated which may be assessed against an accepted cost-effectiveness threshold (national and international).

A discrete-time state-transition Markov model will be used to assess long-term economic impact of the intervention beyond the trial period, with each cycle consisting of relevant events (i.e. maturation/functional patency, failure, complications, re-intervention, alternative access, adverse events, death). Events will be driven by transition probabilities within the model, being informed partly by within-trial data in the short-term (i.e. up to one year) and other sources (literature, electronic health records, etc) in the long-term (i.e. beyond one year).

For peer review only

Retention/withdrawal criteria

Participants may voluntarily withdraw from the study at any time. However, due to the nature of the intervention, it is impossible to change the allocated treatment once the anaesthetic procedure has been performed. Follow-up visits will be timed to coincide with dialysis sessions to minimise follow-up burden and promote trial retention.

Data collection and data management

Study specific data, which is non-identifiable, will be collected on the electronic case report form (eCRF) using a unique patient identifier for reporting. Only the study site will have access to the identifiable information to maintain participant confidentiality. Pseudoanonymised data entered into the eCRF will be managed and stored by the RCB. The RCB systems are fully validated in accordance with industry and regulatory standards and incorporate controlled access security. Data integrity is assured by strictly controlled procedures, including secure data transfer procedures. A computer database will be constructed specifically for the trial data and will include range and logic checks to prevent erroneous data entry. Independent checking of data entry will be periodically undertaken on small sub-samples. The trial statistician will also regularly check the balance of allocations by stratification variables.

All essential documents will be archived in a secure commercial vault for a minimum of 5 years after completion of the trial. Trial data will be stored under controlled conditions for at least 10 years after closure. During this period, all data will be accessible to the competent authorities and the sponsor for audit and monitoring purposes with suitable notice.

Sample size

For peer review only

566 subjects (283 per arm) are required to detect a 15% difference in the primary outcome measure with 5% significance level and 90% power, assuming that 15% of subjects will be lost to follow-up, will change RRT modality, or die.

15% is considered to be the minimum clinically importance difference between the two cohorts. It is a conservative estimate of the 19% difference in one year unassisted functional patency observed in the results from our single-centre RCT and is the magnitude of difference considered appropriate by experts following independent review of the protocol by the UK Renal Trials Network (UKRTN).[12] UK renal registry (UKRR) data indicates that 47% of incident patients currently commence HD via an AVF/AVG.[6] A 15% increase in functional patency would allow the Renal Association target of 60% to be achieved. Similarly, a 15% increase in AVF usage among prevalent HD patients would allow 95% of UK dialysis units to achieve the 80% prevalence target.[7]

Statistical Analysis Plan

All statistical analyses will primarily be performed according to the intention-to-treat principle. However, additional analysis will be pre-specified to address "failed blocks" (e.g. per-protocol, as treated, and complier-average causal effects analyses). Baseline demographics will be summarised by treatment group without formal statistical comparison. The primary outcome will be analysed using logistic regression, adjusting for stratification variables used at randomisation and the treatment group assigned. The treatment effect will be reported with a 95% confidence interval for the Odds Ratio and p-value also reported. Time to loss of functional access will also be analysed using survival analysis regression methods. Similarly, for each of the secondary outcomes, analyses will be

For peer review only

conducted using appropriate regression methods reporting the treatment effects, 95% confidence intervals and p-values. Safety data including the number of adverse events and serious adverse events will be reported overall and by study arm, where no formal statistical testing will be carried out.

Interim analysis and early termination criteria

A 4-month internal pilot will be employed, principally to assess feasibility of recruitment. Stop-go (traffic light) criteria for continuance to the full trial will be used:

- Red: Stop if <50 patients recruited or if <5 centres are open to recruitment
- Amber: Enrol more centres if between 48-95 patients recruited
- Green: Continue within existing parameters if >95 patients recruited

If there is failure of adherence to trial protocol in >20% of participants or significant safety concerns are raised by the IDMC the trial will not progress beyond the pilot phase. In the event that the trial was to be terminated following the internal pilot, all patients would be followed up until the end of trial.

Embedded process evaluation study

An embedded process evaluation study will run in parallel with the trial. The rapid feedback evaluation approach delivered by the Rapid Research Evaluation and Appraisal Lab (RREAL) at the Department of Targeted Intervention, University College London (UCL) will combine qualitative data obtained from semi-structured interviews with patients, carers and staff and documentary analysis (reports, meeting minutes etc.) to:

- Explore staff views and experiences with different approaches to recruitment

For peer review only

- Examine patient and carer experiences in trial participation (understanding of trial literature; experience with treatment options; reasons for withdrawal)
- Examine patient and carer experiences of declining to take part in the trial
- Identify barriers and enablers to trial set-up, recruitment and delivery from the point of view of staff.

Data obtained will be analysed and shared with researchers throughout the main ACCess study at a time when they can be used to inform within trial decision-making processes.[26]

Adverse event reporting

In accordance with the Research Governance Framework for Health and Community Care, any untoward medical occurrence in a trial participant will be considered an adverse event (AE), recorded in the patient's case notes and assessed for severity.[27] Any adverse event that is life-threatening; results in death, birth defect or significant disability; or requires hospitalisation is considered a Serious Adverse Event (SAE). The following trial-specific adverse events will also be considered SAEs:

- A recognised perioperative complication of regional or local anaesthetic administration (including pneumothorax, inadvertent arterial puncture, inadvertent intraneural/intravascular injection, persistent neuropraxia, LA toxicity)
- The requirement for re-exploration or abandonment of surgery

Full details including the nature of the event, start and stop dates, severity, actions taken, relationship to the trial specific intervention and outcome of all SAEs will be reported to the sponsor, via the Glasgow Clinical Trials Unit, on the eCRF and events followed-up until satisfactory resolution. All SAE will be assessed for causality and expectedness. Any SAE believed to be related to a trial specific procedure that is thought to be unexpected (i.e. the

For peer review only

event is not listed within the protocol nor would not be expected to occur when carrying out the trial specific procedure in normal clinical practice) will be considered a Related Unexpected Serious Adverse Event (RUSAE) and must be reported to the sponsor within 24 hours of the site becoming aware. All SAEs will be reported to the IDMC, TSC and sponsor. The sponsor must inform the REC of any RUSAE within 15 days.

Trial management and audit

The TSC, including a patient representative, will provide overall supervision of the trial and ensure that trial conduct is in line with standards set out in the EU Good Clinical Practice (GCP) Guideline.[28] The TSC (including the Chief Investigator, trial statistician and five independent experts) will meet on six occasions during the trial, review blinded safety data biannually and report formally to the sponsor.

An IDMC will be responsible for monitoring data emerging from the trial, in particular as they relate to the safety of participants. The IDMC will be completely independent of the trial and any institutions involved in the trial. It will consist of an expert clinical trialist (chair); expert in the field of vascular access and expert statistician. The IDMC will meet annually during the recruitment and follow-up phases of the trial. The IDMC is the only body that will have access to the unblinded comparative data during the trial. Ultimate responsibility in deciding whether or not to act upon recommendations from the IDMC or a decision for early termination lies with the TSC in conjunction with the sponsor and funder.

For peer review only

Following risk assessment, it has been determined that the study will not be routinely monitored by the sponsor, however the sponsor randomly selects a number of studies to be audited annually. In addition audits can be requested by individual participating sites/TSC.

Public and patient involvement

Patients and the public have been integral to the design and implementation of this trial. Consultation and focus groups identified both the importance of access functionality, which is reflected in our choice of unassisted functional patency as the primary outcome measure, and the "exhaustion and loss of control" experienced by patients on dialysis. The trial protocol reflects these challenges such that follow-up will, where possible, be performed on whilst the patient is on dialysis to minimise the burden additional unnecessary hospital visits and cannulation diaries have been developed so that patients participating in the study will have some ownership for collecting data and reporting outcomes. The embedded process evaluation study will also explore patient and carer experiences of trial participation. A patient representative will sit on the TSC to ensure that the patient's voice is heard throughout the trial. Study participants will receive results via their dialysis units, social media, renal charities and patient groups.

Ethics and dissemination

The trial protocol has been approved by the West of Scotland Research and Ethics Committee (REC) 3 (20/WS/0178). Research will be conducted in accordance with the Declaration of Helsinki, the Principles of Good Clinical Practice, the Data Protection Act (2018), the General Data Protection Regulation and the UK Policy Framework for Health and Social Care Research.

For peer review only

Substantial amendments that require review by REC will not be implemented until the REC grants a favourable opinion for the trial (amendments may also need to be reviewed and approved by the NHS R&D departments before they can be implemented in practice at local sites).

Consent

A member of the research team will obtain written informed consent prior to administration of any trial intervention (Appendix 2). Participants will also be asked to provide consent for future data-linkage studies via the Scottish Renal Registry (SRR) and UK Renal Registry (UKRR). All patients will have the right to refuse participation and withdraw from the trial at any time without providing reasons and without prejudicing further treatment.

Access to data

The anonymised participant level dataset and statistical code of generating the results will be made publicly available within 12 months of the end of trial via an online data repository.

Ancillary and post-trial care

The anaesthetic (both regional and local) reflects a single event intervention, therefore contingency plans for the provision of ongoing treatment for individual trial participants is not required. The sponsor is a member of the Clinical Negligence and Other Risks Indemnity Scheme (CNORIS), which covers the Sponsors legal liability in relation to clinical trials including clinical negligence and harm from study design.

Publication/Dissemination

For peer review only

Ownership of the data arising from this study resides with the grant holders. Research findings will be published in the name of the ACCess Collaborative Group, acknowledging the writing group as authors. Results of this trial will have implications for patients and clinicians across a range of disciplines including nephrology, anaesthesia, vascular access surgery, and dialysis nursing. The principal target audience however is healthcare commissioners and policy makers. Results will be published in open access peer-reviewed journals within 12 months of completion of the trial. We will also present our findings key national/international renal meetings and support dissemination of trial outcomes directly to patients via patient groups, renal charities and social media.

For peer review only

Author affiliations:

1. Department of Anaesthesia, Glasgow Royal Infirmary, Glasgow, UK
2. University of Glasgow, Glasgow, UK
3. Department of Renal Surgery, Queen Elizabeth University Hospital, Glasgow, UK
4. Institute of Cardiovascular and Medical Sciences, University of Glasgow, Glasgow, UK
5. Department of Vascular Surgery, Queen Elizabeth University Hospital, Glasgow, UK
6. Centre for Perioperative Medicine, University College London, London, UK
7. Department of Targeted Intervention, University College London, London, UK
8. Division of Surgery and Interventional Science, University College London, London, UK
9. Independent Health Economist, Healthonomics Ltd, UK
10. Department of Surgery, University of Cambridge, Cambridge, UK
11. Department of Nephrology and Transplantation, Royal Free Hospital, London, UK
12. Department of Transplantation, Guy's and St Thomas' Hospital, London, UK
13. Department of Vascular Surgery, Hairmyres Hospital, East Kilbride, UK
14. Department of Anaesthesia, Freeman Hospital, Newcastle-upon-Tyne, UK
15. Department of Nephrology, Dumfries & Galloway Royal Infirmary, Dumfries, UK
16. Department of Vascular Surgery, Ninewells Hospital, Dundee, UK
17. Robertson Centre of Biostatistics, University of Glasgow, Glasgow, UK
18. Department of Anaesthesia and Perioperative Medicine, Guy's and St Thomas' NHS Foundation Trust, London, UK
19. Honorary Senior Lecturer, King's College London, UK
20. Department of Anaesthesia, Belfast Health and Care Social Care Trust, Belfast, UK
21. Department of Anaesthesia, Queen Elizabeth University Hospital, Glasgow, UK

For peer review only

22. Department of Anaesthesia, Royal Free London NHS Trust, London, UK

Collaborators

Gavin Pettigrew, Regin Lagaac (Addenbrooke's Hospital, Cambridge University Hospitals NHS Foundation Trust), Rosie Hogg (Belfast City Hospital, Belfast Health and Social Care Trust), Stuart Suttie, Andrew Dalton, Samira Bell, Rose Ross (Ninewells Hospital, NHS Tayside), Thalakunte Muniraju, David McNair, Linda Stiff, Catherine Jardine (Dumfries and Galloway Royal Infirmary, NHS Dumfries & Galloway), Rita Singh, Mohammed Tariq Dosani, Jennifer Sainsbury (The Freeman Hospital, Newcastle-upon-Tyne Hospitals NHS Foundation Trust), Nikolaos Karydis, Kiran Sran, Kariem El-Boghdadly, Nadia Castrillo (Guy's and St Thomas' NHS Foundation Trust, London), James Gilbert, Sanjay Sinha, Sheera Sutherland, Sarah Crosbie, Madita Gavrila (Churchill Hospital, Oxford University Hospitals NHS Foundation Trust), Alex Vesey, Sandra Montgomery, Tina McLennan, Nina Tarkowska, Scott Oliver, Liz Brown, Shelley McLachlan (Monklands and Hairmyres Hospitals, NHS Lanarkshire), Jonathan de Siqueira, Max Troxler, Nikki Dewhirst, Mark Wright, Chetan Srinath (Leeds General Infirmary, Leeds Teaching Hospitals NHS Trust), Philip Bennett, Darren Morrow (Norfolk and Norwich University Hospitals NHS Foundation Trust Norwich), Emma Aitken, Marc Clancy, Iain Thomson, Andrew Jackson, Karen Stevenson, David Kingsmore, Margaret Aitken (Queen Elizabeth University Hospital, Glasgow, NHS Greater Glasgow and Clyde), Reza Motallebzadeh, Vishal Nangalia, Vashist Deelchand, Rani Badhan (Royal Free London NHS Trust, London), Rajesh Sivaprakasam, Gareth Ackland Tim Egan, Matt Wikner (The Royal London Hospital, Barts Health NHS Trust), Alan Macfarlane, Rachel Kearns (Stobhill Ambulatory Care Hospital, Glasgow, NHS Greater Glasgow and Clyde), Lucian Gaianu (Independent Health Economist), Alex MacConnachie, Kirsty Wetherall,

For peer review only

Patrick Mark (University of Glasgow), Ramani Moonsinghe, Cecilia Vindrola (University College London).

Authors' contributions

AJRM, RJK, MC, DBK, KS, AJ, PM, MA, RM, CV, LG, GJP, RM, NK, AV, RS, TM, SS, AMcC, KW, KE, RH, IT, EA contributed to the development and implementation of this protocol and have approved the manuscript. The authors would also like to acknowledge the contribution of Ewen Maclean, patient and public involvement representative for trial design and implementation and Chloe Knott, patient representative on the TSC.

Funding

This work is supported by National Institute for Health Research (NIHR) [Health Technology Assessment (HTA) 130567]. The views expressed are those of the authors and not necessarily those of the NIHR or the Department of Health and Social Care.

Competing interests

AJRM is currently President-elect of Regional Anaesthesia UK (RA-UK). RA-UK has reviewed and endorsed the trial protocol, but has not been involved in the design or development in any way. AJRM has received consulting fees from Heron therapeutics and intelligent US. RH has received honoraria from GE.

Sponsor

The trial sponsor is NHS Greater Glasgow and Clyde

For peer review only

Sponsor's Representative

Dr Maureen Travers, Senior R&D Co-ordinator, NHS Greater Glasgow and Clyde Email:

Maureen.Travers@ggc.scot.nhs.uk

Project Manager

Lisa Jolly, R&D Project Manager, NHS Greater Glasgow and Clyde. Email:

Lisa.Jolly@ggc.scot.nhs.uk

Patient consent for publication

Not required

Twitter

@study_access

For peer review only

References

1. Scottish Renal Registry (2018) Annual Report. Available online at: https://www.srr.scot.nhs.uk/ Publications/docs/2018-10-09-SRR-Report.pdf?4.
2. USRDS (2014). USRDS Annual Data Report 2014: Volume 2, End stage renal disease in the United States. https://www.usrds.org/2013/pdf/v2_esrd_full_13.pdf.
3. National Kidney Care Healthcare Quality Improvement Partnership (2011) National Kidney Care Vascular Access Audit Report 2011
4. Wasse H, Kutner N, Zhang R & Huang Y. Association of Initial Haemodialysis Vascular Access with Patient-Reported Health Status and Quality of Life. *Clin J Am Soc Nephrol* 2007*;* **2**: 708-14.
5. Malas MB, Canner JK, Hicks CW et al.Trends in incident vascular access and mortality. *JAMA Surgery* 2015; **150**: 441-8.
6. UK Renal Registry (2017) 20th Annual Report. https://www.renalreg.org/reports/2017-twentieth-annual-report
7. Kumwenda M, Mitra S & Reid C (2017) Clinical Practice Guideline: Vascular Access for Haemodialysis https://renal.org/sites/renal.org/files/vascular-access.pdf
8. Aitken E, Jackson A, Kearns R et al. Effect of regional vs local anaesthesia on outcome after arteriovenous fistula creation: randomised controlled trial. *Lancet* 2016; **388**:1067-74.
9. Dember L, Beck G, Allon M et al. Effect of clopidogrel on early failure of arteriovenous fistulas for hemodialysis: randomised controlled trial. *JAMA* 2008*;* **299**: 2164-71.
10. Keuler J, Weiss C, Klemm K et al. Assessing changes in tissue oxygenation by near infrared spectroscopy following brachial plexus block for arteriovenous fistula surgery: A prospective observational pilot study. *Eur J Anaesthesiol* 2018*;* **35**: 759-65.
11. Cerneviciute R, Sahebally M, Ahmed K et al. Regional versus local anaesthesia for haemodialysis arteriovenous fistula formation: systematic review and meta-analysis *Eur J Vasc Endovasc Surg* 2017; **53**: 34-42.
12. Aitken E, Kearns R, Gaianu L, et al. Long-term functional patency and cost-effectiveness of arteriovenous fistula creation under regional anaesthesia: a randomised controlled trial *J Am Soc Nephrol* 2020*;* **31**: 1871-82.
13. Schmidli J , Widmer MW, Basile C et al. Vascular Access: 2018 Clinical Practice Guidelines of the European Society for Vascular Surgery*. Eur J Vasc Endovasc Surg* 2018; **55**: e757-e818.
14. ERA-EDTA. Clinical Practice Guideline on peri- and post-operative care of arteriovenous fistulas and grafts for haemodialysis in adults. http://www.european-renal-best-practice.org/sites/defailut/files/u33/ERBP_006-VASCAC.ShortVersion.pdf.
15. Lok CE, Huber TS, Lee T et al. KDOQI Clinical Practice Guidelines for Vascular Access:2019 update. *Am J Kid Dis* 2020; **75**: S1-164.

16. Armstrong RA, Wilson C, Elliot L et al. Regional anaesthesia for arteriovenous fistula formation surgery. *Anaesthesia* 2020; **75**: 626-33.
17. Bradley T, Teare T & Milner Q. Anaesthetic management of patients requiring vascular access surgery for renal dialysis. *BJA Education* 2017*;* **17**: 269-74.
18. Horlocker TT, Vandermeulen E, Kopp SI et al. Regional anaesthesia in the patient receiving antithrombotic or thrombolytic therapy *Reg Anes Pain Med* 2018; **43**: 263-309.
19. Kant A, Gupta PK, Zohar S, Chevret S, Hopkins PM. Application of the continual reassessment method to dose-finding studies in regional anesthesia: an assessment of the ED95 dose for 0.5 bupivacaine for ultrasound-guided supraclavicular block. *Anesthesiology* 2013; **119**: 29-35.
20. Gadsden J and Orebaugh S. Targeted intracluster supraclavicular brachial plexus block: too close for comfort. *Br J Anaesth* 2019*;* **122**: 713-5.
21. Rodriguez J, Taboada-Muniz M, Barcena M, Alvarez J Median versus musculocutaneous nerve response with single-injection infraclavicular coracoid block. *Reg Anesth Pain Med* 2014*;* **29**: 534–8.
22. Beathard GA, Lok CE, Glickman MH et al. Definitions and End Points for Interventional Studies for Arteriovenous Dialysis Access. *Clin J Am Soc Nephrol* 2018; **13**: 501-12.
23. Robbins M, Chamberlain N, Lockhart M et al. Hemodialysis arteriovenous fistula maturity: US evaluation. *Radiology* 2002; **225**: 59-64.
24. Verberne WR, Das-Gupta Z, Allegretti A et al. Development of an International Standard Set of Value-Based Outcome Measures for Patients with Chronic Kidney Disease: A report of the ICHOM CKD Working Group. *Am J Kidney Dis* 2019*;* **73**: 372-84.
25. NICE. Interventional procedure overview of ultrasound-guided regional nerve block. Available online at: https://www.nice.org.uk/guidance/IPG285
26. McNall M, Welch V, Ruh K et al. (2004) The use of rapid-feedback evaluation methods to improve the retention rates of an HIV/AIDS healthcare intervention. *Evaluation and Program Planning;* 27: 287-294.
27. UK policy framework for health and social care research. NHS Health research Authority 2020. https://s3.eu-west-2.amazonaws.com/www.hra.nhs.uk/media/documents/Final_Accessibility_uk-policy-framework-health-social-care-research_.pdf
28. International Conference on Harmonization (ICH) guideline on Good Clinical Practice (1996) http://www.ich.org/products/guidelines/efficacy/efficacy-single/article/good-clinical-practice.html
29. Brimble KS, Rabbat C, Treleaven DJ, Ingram AJ. Utility of ultrasonographic venous assessment prior to forearm arteriovenous fistula creation. *Clin Nephrol* 2002*;* **58**: 122-7.
30. AAGBI, Obstetric Anaesthetists Association and RA-UK (2013) Regional anaesthesia and patients with abnormalities of coagulation. *Anaesthesia* 2013*;* **68**: 966-72.
31. ED-5D User Guide, EuroQOL Group. Available online at: https://euroqol.org/wp-content/uploads/2016/09/EQ-5D-5L_UserGuide_2015.pdf

For peer review only

Kidney Disease Quality of Life Short Form (KDQOL-SF). The Rand Corporation. Available online at: https://www.rand.org/health-care/surveys_tools/kdqol.html

For peer review only

Box 1. Exclusion criteria.

General

- Patients unable or unwilling to provide informed consent
- Patient preference for general or alternative anaesthesia
- Active infection at surgical or anaesthetic site

Access specific

- Previous ipsilateral AVF creation (a previous attempt at distal AVF creation which fails immediately is not considered a contraindication, however any distal access which has previously run sufficiently to mature the outflow vein or proximal revision of an existing AVF is considered a contraindication)
- Known ipsilateral cephalic arch or central venous stenosis (even if previously treated)
- USS evidence of stenosis in inflow artery
- Radial or brachial artery <1.8mm diameter and/or cephalic vein <2mm at wrist or <3mm at elbow (with tourniquet) on pre-operative USS[29]

Contraindications to anaesthetic agents/ technique:

- Allergy to LA or any excipient agents
- Acquired or inherited coagulopathy (including warfarin/ heparin/ novel oral anticoagulant use where it has not been possible to stop the anticoagulation in anticipation of surgery) and/or platelets <75 or INR > 1.4[30]
- Significant pre-existing neurological disorder affecting upper limb
- Weight <45kg

For peer review only

Box 2: Secondary outcome measures

Access specific outcome measures

- Patency (i.e. is the fistula running ?): defined clinically as the presence of a bruit
- Access complications (including infection, stenosis, thrombosis, steal, bleeding)
- Re-operation/re-intervention to maintain or re-establish patency (revisional surgery, angioplasty, stenting or thrombectomy)
- Alternative accesses e.g. CVCs
- Time to first cannulation
- Cannulation difficulties (including failure to establish two needle dialysis, infiltration, prolonged bleeding)

Patient specific outcome measures:

- Mortality
- Date commenced on HD
- Access modality at start of HD
- Change of RRT modality
- Change of access modality
- Access-related hospitalisation

HR-QoL:

- EQ-5D-5L (EuroQol)[31]
- Kidney Disease Quality of Life Short Form (KDQOL-SF)[32]
- Vascular Access Specific Quality Of Life (VASQOL)

Safety outcome measures:

- Adverse events relating to anaesthesia e.g. systemic toxicity, pneumothorax, nerve damage, intravascular injection
- Technical difficulties delivering anaesthesia e.g. inability to identify structures, misplacement, paraesthesia

Anaesthesia:

- Pain score at incision, at 30 minutes and 1 hour postoperatively (NRS 0-10)
- Speed of onset/quality of motor and sensory block[21]
- Need for anaesthetic supplementation
- ”Failed block”
- Volume of anaesthetic agent (mL)
- Time to administer anaesthetic (mins)

Other:

- Change in surgical plan e.g. switch from BCF to RCF prior to surgical incision

For peer review only

Figure 1. Participant timeline.

Appendix 1. Preparation of anaesthetic mixture for RA and administration of brachial plexus block.

Appendix 2. Consent form.

For peer review only

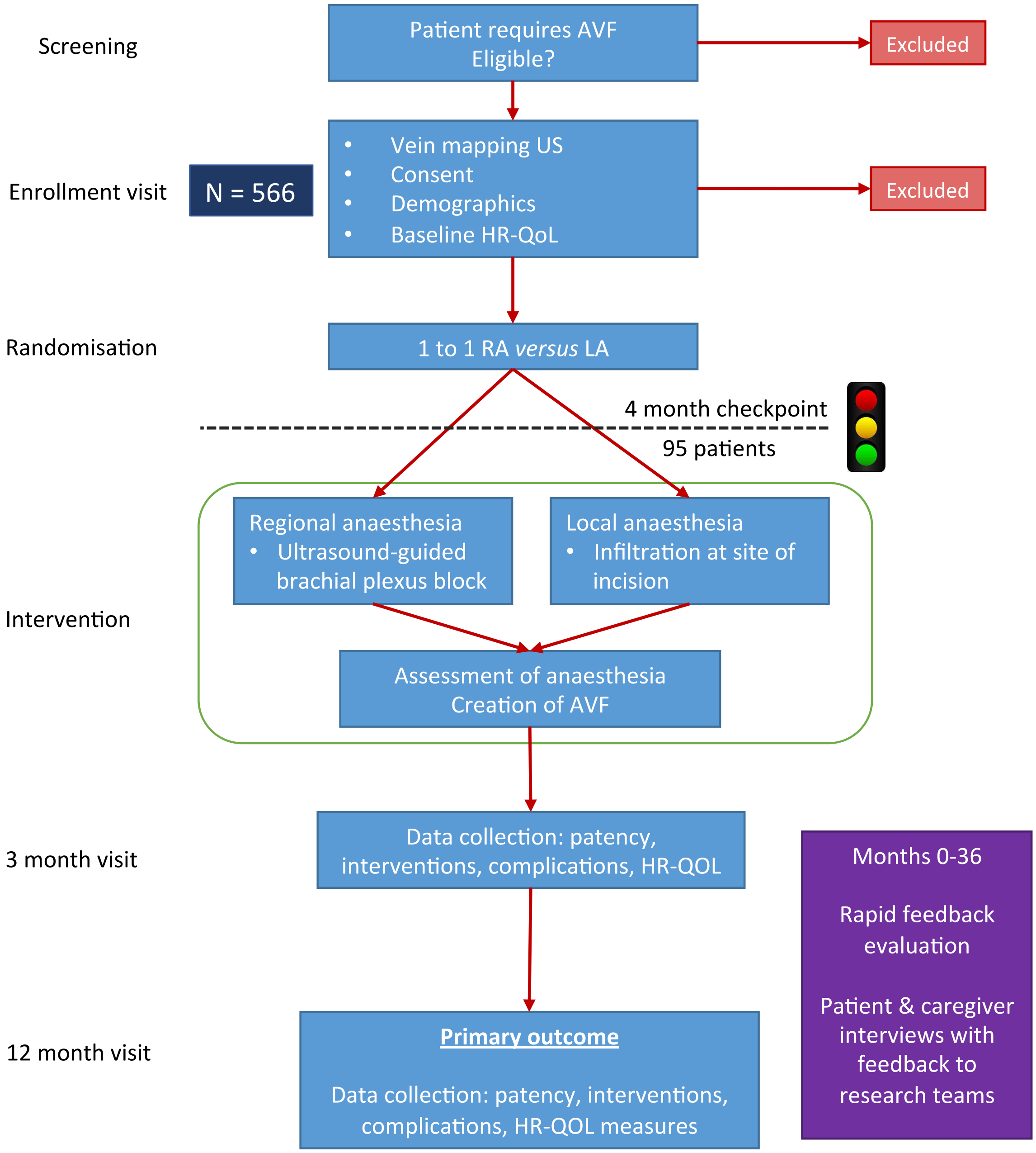
Screening
Patient requires AVF
Eligible?
Excluded
Enrollment visit
N = 566
Vein mapping US
Consent
Demographics
Baseline HR-QoL
Excluded
Randomisation
1 to 1 RA versus LA
4 month checkpoint
95 patients
Intervention
Regional anaesthesia
Ultrasound-guided brachial plexus block
Local anaesthesia
Infiltration at site of incision
Assessment of anaesthesia
Creation of AVF
3 month visit
Data collection: patency, interventions, complications, HR-QOL
Months 0-36
Rapid feedback evaluation
Patient & caregiver interviews with feedback to research teams
12 month visit
Primary outcome
Data collection: patency, interventions, complications, HR-QOL measures

Appendix 1. Preparation of anaesthetic mixture for RA and administration of brachial plexus block.

Preparation:
Prepare 2 x 20ml syringes.
To each syringe add:

- 10ml 0.5% Levobupivacaine
- 10ml 1% Lidocaine
- 0.05ml 1:1,000 Adrenaline

Administration of brachial plexus block:
A minimum volume based on weight (outlined below) must be injected during the initial block (whether supraclavicular or axillary).

- 45-50kg: 15ml
- 51-60kg: 20ml
- >60kg: 25ml

Larger volumes may be used at the discretion of the anaesthetist as long as maximum dose limits are observed, remembering that local anaesthetic may also be required for surgical supplementation and that these doses are additive. Consider using ideal body weight in obese patients.

For peer review only

ACCESS

Anaesthesia Choice for Creation of ArtEriovenous FiStulae (ACCess study)

Chief Investigator: Emma Aitken

Participant Identification Number:

Please initial each box if you agree with the following statements:

I confirm that I have read and understood the information sheet dated *XX/XX/XX* (version *X.X)* for the above study. I have had the opportunity to consider the information, ask questions and have had those questions answered satisfactorily.	
I understand that my participation is voluntary and that I am free to withdraw at any time without giving any reason, without my medical care or legal rights being affected.	
I understand that relevant sections of my medical notes and data collected during the study may be looked at by individuals from the research team, regulatory authorities or from the Sponsor, NHS Greater Glasgow and Clyde, where it is relevant to my taking part in the research. I give permission to these individuals to have access to my records. I understand that my data will be held by the University of Glasgow.	
I understand that the information collected about me will be used to support other research in the future, and may be shared anonymously with other researchers.	
I agree to the study team having my phone number for the purpose of contacting me during the study.	
I agree to take part in the above study.	

Name of Participant **Signature** **Date**

………………………………… ………………………………… ……………………

Name of Researcher **Signature** **Date**

………………………………… ………………………………… ……………………

When completed: 1 for participant; 1 for researcher site file; 1 to be kept in medical notes.

For peer review only - http://bmjopen.bmj.com/site/about/guidelines.xhtml

SPIRIT 2013 Checklist: Recommended items to address in a clinical trial protocol and related documents*

Section/item	Item No	Description
**Administrative information**		
Title	1 Y	Descriptive title identifying the study design, population, interventions, and, if applicable, trial acronym
Trial registration	2a Y	Trial identifier and registry name. If not yet registered, name of intended registry
	2b Y	All items from the World Health Organization Trial Registration Data Set
Protocol version	3 Y	Date and version identifier
Funding	4 Y	Sources and types of financial, material, and other support
Roles and responsibilities	5a Y	Names, affiliations, and roles of protocol contributors
	5b Y	Name and contact information for the trial sponsor
	5c Y	Role of study sponsor and funders, if any, in study design; collection, management, analysis, and interpretation of data; writing of the report; and the decision to submit the report for publication, including whether they will have ultimate authority over any of these activities
	5d Y	Composition, roles, and responsibilities of the coordinating centre, steering committee, endpoint adjudication committee, data management team, and other individuals or groups overseeing the trial, if applicable (see Item 21a for data monitoring committee)
**Introduction**		
Background and rationale	6a Y	Description of research question and justification for undertaking the trial, including summary of relevant studies (published and unpublished) examining benefits and harms for each intervention
	6b Y	Explanation for choice of comparators
Objectives	7 Y	Specific objectives or hypotheses
Trial design	8 Y	Description of trial design including type of trial (eg, parallel group, crossover, factorial, single group), allocation ratio, and framework (eg, superiority, equivalence, noninferiority, exploratory)
**Methods: Participants, interventions, and outcomes**		
Study setting	9 Y	Description of study settings (eg, community clinic, academic hospital) and list of countries where data will be collected. Reference to where list of study sites can be obtained

For peer review only

Eligibility criteria	10 Y	Inclusion and exclusion criteria for participants. If applicable, eligibility criteria for study centres and individuals who will perform the interventions (eg, surgeons, psychotherapists)
Interventions	11aY	Interventions for each group with sufficient detail to allow replication, including how and when they will be administered
	11bY	Criteria for discontinuing or modifying allocated interventions for a given trial participant (eg, drug dose change in response to harms, participant request, or improving/worsening disease)
	11cY	Strategies to improve adherence to intervention protocols, and any procedures for monitoring adherence (eg, drug tablet return, laboratory tests)
	11dY	Relevant concomitant care and interventions that are permitted or prohibited during the trial
Outcomes	12 Y	Primary, secondary, and other outcomes, including the specific measurement variable (eg, systolic blood pressure), analysis metric (eg, change from baseline, final value, time to event), method of aggregation (eg, median, proportion), and time point for each outcome. Explanation of the clinical relevance of chosen efficacy and harm outcomes is strongly recommended
Participant timeline	13 Y	Time schedule of enrolment, interventions (including any run-ins and washouts), assessments, and visits for participants. A schematic diagram is highly recommended (see Figure)
Sample size	14 Y	Estimated number of participants needed to achieve study objectives and how it was determined, including clinical and statistical assumptions supporting any sample size calculations
Recruitment	15 Y	Strategies for achieving adequate participant enrolment to reach target sample size
**Methods: Assignment of interventions (for controlled trials)**		
Allocation:		
Sequence generation	16aY	Method of generating the allocation sequence (eg, computer-generated random numbers), and list of any factors for stratification. To reduce predictability of a random sequence, details of any planned restriction (eg, blocking) should be provided in a separate document that is unavailable to those who enrol participants or assign interventions
Allocation concealment mechanism	16bY	Mechanism of implementing the allocation sequence (eg, central telephone; sequentially numbered, opaque, sealed envelopes), describing any steps to conceal the sequence until interventions are assigned
Implementation	16cY	Who will generate the allocation sequence, who will enrol participants, and who will assign participants to interventions
Blinding (masking)	17aY	Who will be blinded after assignment to interventions (eg, trial participants, care providers, outcome assessors, data analysts), and how

	17bY	If blinded, circumstances under which unblinding is permissible, and procedure for revealing a participant's allocated intervention during the trial
**Methods: Data collection, management, and analysis**		
Data collection methods	18aY	Plans for assessment and collection of outcome, baseline, and other trial data, including any related processes to promote data quality (eg, duplicate measurements, training of assessors) and a description of study instruments (eg, questionnaires, laboratory tests) along with their reliability and validity, if known. Reference to where data collection forms can be found, if not in the protocol
	18bY	Plans to promote participant retention and complete follow-up, including list of any outcome data to be collected for participants who discontinue or deviate from intervention protocols
Data management	19 Y	Plans for data entry, coding, security, and storage, including any related processes to promote data quality (eg, double data entry; range checks for data values). Reference to where details of data management procedures can be found, if not in the protocol
Statistical methods	20aY	Statistical methods for analysing primary and secondary outcomes. Reference to where other details of the statistical analysis plan can be found, if not in the protocol
	20bY	Methods for any additional analyses (eg, subgroup and adjusted analyses)
	20cY	Definition of analysis population relating to protocol non-adherence (eg, as randomised analysis), and any statistical methods to handle missing data (eg, multiple imputation)
**Methods: Monitoring**		
Data monitoring	21aY	Composition of data monitoring committee (DMC); summary of its role and reporting structure; statement of whether it is independent from the sponsor and competing interests; and reference to where further details about its charter can be found, if not in the protocol. Alternatively, an explanation of why a DMC is not needed
	21bY	Description of any interim analyses and stopping guidelines, including who will have access to these interim results and make the final decision to terminate the trial
Harms	22Y	Plans for collecting, assessing, reporting, and managing solicited and spontaneously reported adverse events and other unintended effects of trial interventions or trial conduct
Auditing	23Y	Frequency and procedures for auditing trial conduct, if any, and whether the process will be independent from investigators and the sponsor
**Ethics and dissemination**		
Research ethics approval	24Y	Plans for seeking research ethics committee/institutional review board (REC/IRB) approval

Protocol amendments	25Y	Plans for communicating important protocol modifications (eg, changes to eligibility criteria, outcomes, analyses) to relevant parties (eg, investigators, REC/IRBs, trial participants, trial registries, journals, regulators)
Consent or assent	26aY	Who will obtain informed consent or assent from potential trial participants or authorised surrogates, and how (see Item 32)
	26bY	Additional consent provisions for collection and use of participant data and biological specimens in ancillary studies, if applicable
Confidentiality	27Y	How personal information about potential and enrolled participants will be collected, shared, and maintained in order to protect confidentiality before, during, and after the trial
Declaration of interests	28Y	Financial and other competing interests for principal investigators for the overall trial and each study site
Access to data	29Y	Statement of who will have access to the final trial dataset, and disclosure of contractual agreements that limit such access for investigators
Ancillary and post-trial care	30Y	Provisions, if any, for ancillary and post-trial care, and for compensation to those who suffer harm from trial participation
Dissemination policy	31aY	Plans for investigators and sponsor to communicate trial results to participants, healthcare professionals, the public, and other relevant groups (eg, via publication, reporting in results databases, or other data sharing arrangements), including any publication restrictions
	31bY	Authorship eligibility guidelines and any intended use of professional writers
	31cY	Plans, if any, for granting public access to the full protocol, participant-level dataset, and statistical code
**Appendices**		
Informed consent materials	32Y	Model consent form and other related documentation given to participants and authorised surrogates
Biological specimens	33 N/A	Plans for collection, laboratory evaluation, and storage of biological specimens for genetic or molecular analysis in the current trial and for future use in ancillary studies, if applicable

*It is strongly recommended that this checklist be read in conjunction with the SPIRIT 2013 Explanation & Elaboration for important clarification on the items. Amendments to the protocol should be tracked and dated. The SPIRIT checklist is copyrighted by the SPIRIT Group under the Creative Commons "Attribution-NonCommercial-NoDerivs 3.0 Unported" license.

For peer review only

BMJ Open

Anaesthesia Choice for Creation of arteriovEnouS fiStula (ACCess) study protocol : a randomised controlled trial comparing primary unassisted patency at one year of primary arteriovenous fistulae created under regional compared to local anaesthesia

Journal:	*BMJ Open*
Manuscript ID	bmjopen-2021-052188.R1
Article Type:	Protocol
Date Submitted by the Author:	14-Sep-2021
Complete List of Authors:	Macfarlane, Alan; NHS Greater Glasgow and Clyde, Department of Anaesthesia; University of Glasgow, Department of Anaesthesia<br>Kearns, Rachel J; NHS Greater Glasgow and Clyde, Department of Anaesthesia; University of Glasgow, Department of Anaesthesia<br>Clancy , Marc James; Queen Elizabeth University Hospital, Department of Renal Surgery; University of Glasgow, Institute of Cardiovascular and Medical Sciences<br>Kingsmore, David; Queen Elizabeth University Hospital, Department of Vascular Surgery; University of Glasgow, Institute of Cardiovascular and Medical Sciences<br>Stevenson, Karen; Queen Elizabeth University Hospital, Department of Renal Surgery<br>Jackson, Andrew; Queen Elizabeth University Hospital, Department of Renal Surgery<br>Mark, Patrick; University of Glasgow, Institute of Cardiovascular & Medical Sciences; Queen Elizabeth University Hospital Campus, Department of Nephrology<br>Aitken, Margaret; Queen Elizabeth University Hospital, Department of Renal Surgery<br>Moonsinghe, Ramani; University College London, Centre for Perioperative Medicine; University College London Hospitals NHS Foundation Trust, Anaesthesia and Critical Care<br>Vindrola-Padros, Cecilia; University College London, Rapid Research Evaluation and Appraisal Lab<br>Gaianu, Lucian; Independent Health Economist, Healthonomics UK Ltd<br>Pettigrew, Gavin ; Cambridge University, Surgery; Addenbrooke's Hospital, Department of Surgery<br>Motallebzadeh, Reza; Royal Free London NHS Foundation Trust, Department of Nephrology and Transplantation; University College London, Department of Surgery and Interventional Science<br>Karydis, Nikolaos; Guy's and St Thomas' NHS Foundation Trust, Department of Transplantation<br>Vesey, Alex; University Hospital Hairmyres, Department of Vascular Surgery<br>Singh, Rita; Freeman Hospital, Department of Anaesthesia<br>Muniraju, Thalakunte; Dumfries and Galloway Acute Hospitals, Department of Nephrology

	Suttie, Stuart; Ninewells Hospital and Medical School, Department of Vascular Surgery<br>McConnachie, Alex; University of Glasgow, Robertson Centre for Biostatistics<br>Wetherall, Kirsty; University of Glasgow, Robertson Centre for Biostatistics<br>El-Boghdadly, Kariem; Guy's and Saint Thomas' NHS Foundation Trust, Department of Anaesthesia and Perioperative Medicine; King's College London<br>Hogg, Rosemary; Belfast Health and Social Care Trust, Department of Anaesthesia<br>Thomson, Iain; Queen Elizabeth University Hospital, Department of Anaesthesia<br>Nangalia, Vishal; Royal Free London NHS Foundation Trust, Department of Anaesthesia<br>Aitken, Emma; Queen Elizabeth University Hospital, Department of Renal Surgery; University of Glasgow, Institute of Cardiovascular & Medical Sciences
**Primary Subject Heading**:	Anaesthesia
Secondary Subject Heading:	Health economics, Renal medicine, Surgery
Keywords:	Adult anaesthesia < ANAESTHETICS, End stage renal failure < NEPHROLOGY, Vascular surgery < SURGERY, Transplant surgery < SURGERY

SCHOLARONE™
Manuscripts

Title :

Anaesthesia Choice for Creation of arteriovEnouS fiStula (ACCess) study protocol : a randomised controlled trial comparing primary unassisted patency at one year of primary arteriovenous fistulae created under regional compared to local anaesthesia.

Authors :

Alan JR Macfarlane[1,2], Rachel J Kearns[1,2], Marc Clancy[3,4], David Kingsmore[4,5], Karen Stevenson[3], Andrew Jackson[3], Patrick B Mark[4], Margaret Aitken[3], Ramani Moonesinghe[6,7,8], Cecilia Vindrola-Padros[7], Lucian Gaianu[9], Gavin Pettigrew[10], Reza Motallebzadeh[8,11], Nikolaos Karydis[12], Alex Vesey[13], Rita Singh[14], Thalakunte Muniraju[15], Stuart Suttie[16], Alex McConnachie[17], Kirsty Wetherall[17], Kariem El Boghdadly[18,19], Rosemary Hogg[20], Iain Thomson[21], Vishal Nangalia[22], Emma Aitken[3,4]. On behalf of the ACCess collaborative group.

Author affiliations:

1. Department of Anaesthesia, Glasgow Royal Infirmary, Glasgow, UK
2. University of Glasgow, Glasgow, UK
3. Department of Renal Surgery, Queen Elizabeth University Hospital, Glasgow, UK
4. Institute of Cardiovascular and Medical Sciences, University of Glasgow, Glasgow, UK
5. Department of Vascular Surgery, Queen Elizabeth University Hospital, Glasgow, UK
6. Centre for Perioperative Medicine, University College London, London, UK
7. Department of Targeted Intervention, University College London, London, UK
8. Division of Surgery and Interventional Science, University College London, London, UK
9. Independent Health Economist, Healthonomics Ltd, UK

For peer review only

10. Department of Surgery, University of Cambridge, Cambridge, UK

11. Department of Nephrology and Transplantation, Royal Free Hospital, London, UK

12. Department of Transplantation, Guy's and St Thomas' Hospital, London, UK

13. Department of Vascular Surgery, Hairmyres Hospital, East Kilbride, UK

14. Department of Anaesthesia, Freeman Hospital, Newcastle-upon-Tyne, UK

15. Department of Nephrology, Dumfries & Galloway Royal Infirmary, Dumfries, UK

16. Department of Vascular Surgery, Ninewells Hospital, Dundee, UK

17. Robertson Centre of Biostatistics, University of Glasgow, Glasgow, UK

18. Department of Anaesthesia and Perioperative Medicine, Guy's and St Thomas' NHS Foundation Trust, London, UK

19. Honorary Senior Lecturer, King's College London, UK

20. Department of Anaesthesia, Belfast Health and Care Social Care Trust, Belfast, UK

21. Department of Anaesthesia, Queen Elizabeth University Hospital, Glasgow, UK

22. Department of Anaesthesia, Royal Free London NHS Trust, London, UK

Corresponding author :

Professor Alan Macfarlane

Consultant Anaesthetist

Glasgow Royal Infirmary, 84 Castle Street, Glasgow, G4 0SF, UK

Honorary Professor, University of Glasgow, Glasgow, UK

Telephone: +44 (0)141 201 3870

Email: alan.macfarlane@glasgow.ac.uk

For peer review only

Word Count : 3936

For peer review only

For peer review only - http://bmjopen.bmj.com/site/about/guidelines.xhtml

Abstract

Introduction

Arteriovenous fistulae (AVF) are the “gold standard” vascular access for haemodialysis. Universal usage is limited however by a high early failure rate. Several small, single-centre studies have demonstrated better early patency rates for AVF created under regional anaesthesia compared to local anaesthesia. The mechanistic hypothesis is that the sympathetic blockade associated with regional anaesthesia causes vasodilatation and increased blood flow through the new AVF. Despite this, considerable variation in practice exists in the UK. A high quality, adequately powered, multicentre randomised controlled trial (RCT) is required to definitively inform practice.

Methods and analysis

The Anaesthesia Choice for Creation of arteriovEnouS fiStula (ACCess) study is a multicentre, observer-blinded, RCT comparing primary radio-/brachio-cephalic AVF created under regional versus local anaesthesia. The primary outcome is primary unassisted AVF patency at one year. Access-specific (e.g. stenosis/ thrombosis), patient-specific (including health-related quality of life (HRQoL)) and safety secondary outcomes will be evaluated. Health economic analysis will also be undertaken.

Ethics and dissemination

The ACCess study has been approved by the West of Scotland Research and Ethics Committee Number 3 (20/WS/0178). Results will be published in open access peer-reviewed journals within 12 months of completion of the trial. We will also present our

For peer review only

findings at key national and international renal and anaesthetic meetings, and support dissemination of trial outcomes via renal patient groups.

Registration details

Trial registration number : ISRCTN14153938 (registered: 11th November 2020)

Sponsor: NHS Greater Glasgow and Clyde GN19RE456; Protocol Version 1.3 (8th May 2021)

REC/IRAS ID: 290482

Strengths and limitations of this study

- This is a prospective, multicentre randomised, observer blinded trial designed to examine whether primary radio-/brachio-cephalic arteriovenous fistulae created under regional anaesthesia rather than local anaesthesia have better one-year primary unassisted functional patency.
- With 566 participants, this will be the largest trial to date comparing regional to local anaesthesia and will address criticisms of previous smaller, single centre randomised trials.
- An associated cost-effectiveness analysis will provide sufficient evidence to guide practice and policy.
- The main limitation of this study is that in pre-dialysis patients, the primary endpoint uses surrogate markers of fistula patency (clinical assessment and ultrasound (USS) criteria) rather than successful use of the fistula for haemodialysis.

For peer review only

Introduction

The incidence of kidney failure requiring kidney replacement therapy (KRT) has increased substantially over the last 30 years and over 25,000 people in the UK are currently treated with maintenance haemodialysis (HD).[1] Kidney disease has a significant impact on both longevity and quality of life and places considerable demand on healthcare resources.[2] Vascular access is "a major modifiable risk factor" in terms of patient experience and outcome on HD, with arteriovenous fistulae (AVF) being the preferred mode of vascular access.[3] Patients dialysing via AVF experience less infective and thrombotic complications, and are three times less likely to be admitted to hospital than their counterparts with central venous catheters (CVCs).[3] Such frequent hospitalisations have a negative impact on health-related quality of life (HRQoL).[4] Furthermore, there is observational data demonstrating superior survival in patients successfully dialysing via AVF compared to those using central venous catheters (CVCs) or prosthetic arteriovenous grafts (AVGs) for dialysis.[5]

Despite these benefits, universal adoption of AVF remains suboptimal. The most recent UK Renal Registry (UKRR) Multisite Dialysis Access Audit highlighted that nearly 80% of dialysis units in England, Wales and Northern Ireland still fail to achieve Renal Association targets which recommend 60% of incident patients receive HD via AVF or AVG.[6,7] One principal obstacle to the widespread utilisation of AVF is "failure to mature", with early failure rates approaching 50%.[8,9] Any intervention that improved AVF maturation should confer significant benefit to patient health and wellbeing, reduce surgical workload and deliver cost savings.

For peer review only

Anaesthetic technique is one such factor believed to influence AVF maturation and outcome. Regional anaesthesia (RA), unlike local anaesthesia (LA), generates a sympathetic blockade. The mechanistic hypothesis is this sympathetic blockade results in vasodilatation, improved tissue oxygenation and increased blood flow through the new AVF therefore reducing early thrombosis.[8,10] Several studies have demonstrated superior short-term patency rates of AVF created under brachial plexus block (BPB) compared to LA.[8,11] In the only RCT to date with prolonged follow-up, RA improved both immediate and one-year functional AVF patency compared to LA.[12] A concomitant health economic analysis using HRQoL data extrapolated from the literature established net cost savings at one-year and an incremental cost-effectiveness ratio of approximately £12,900 per quality-adjusted life year (QALY) gained over a 5-year time horizon with RA.

Both European Society for Vascular Surgery and European Renal Association guidelines suggest considering using RA for all primary AVF.[13,14] The Kidney Disease Outcomes Quality Initiative Vascular Access guidelines disagree, stating choice of anaesthesia should be based on operator discretion.[15] Such disparity regarding the choice of anaesthesia for AVF creation is reflected across UK centres, with significant variation in practice.[16] Whilst this is in part due to a lack of anaesthetic availability or capability, the failure to modify local practices perhaps also reflects the lack of strong evidence. All RCTs to date have been single-centre, with some suffering from methodological flaws.[11] Further, more robust, health economics analysis is required to establish whether any potential durable clinical benefit could be offset against the longer procedural times, need for a skilled anaesthetist and the additional upfront costs associated with RA. Only a definitive, adequately powered, multicentre RCT with associated cost-effectiveness analysis will provide sufficient evidence to change

For peer review only

practice and policy. The ACCess trial aims to address this issue, with the primary objective being to determine whether or not the sympathetic blockade associated with regional compared to local anaesthesia translates into clinical improvements in long term functional fistula patency.

Methods and Analysis

The ACCess study is a multicentre, observer-blinded, parallel group, superiority RCT with an internal pilot and embedded process evaluation study comparing patients undergoing primary radio- (RCF) or brachio-cephalic fistula (BCF) creation under RA versus LA. The primary outcome is unassisted functional AVF patency at one year. The participant timeline is outlined in Figure 1.

Participants

Patients will be recruited from high (>150 cases per year) and medium (>50 cases per year) volume UK centres providing vascular access for HD.

Recruitment

Potentially eligible participants will be identified from vascular access and/or 'pre-dialysis' (low clearance) clinics and theatre waiting lists by the clinical team. A vein mapping ultrasound will be performed to ensure minimum vessel characteristics. We anticipate 12-20 centres recruiting an average of 2 patients/centre/month. Recruitment is due to commence May 2021 and is anticipated to take two-years. Preliminary results are expected late 2024.

For peer review only - http://bmjopen.bmj.com/site/about/guidelines.xhtml

Inclusion criteria

Inclusion criteria are outlined in Box 1.

Exclusion criteria

Exclusion criteria are outlined in Box 1.

Allocation

A central randomisation facility (interactive web response system, IWRS) at the Robertson Centre for Biostatistics (RCB), University of Glasgow, will randomise patients 1:1 to the intervention group (RA) or comparator group (LA). The randomisation list will be created by a computer-generated program, using a method of permuted blocks stratified by centre, dialysis status (pre-dialysis/HD) and site of AVF (RCF/BCF). Randomisation will take place at a patient, not centre, level to minimise bias from variation in surgical and dialysis practice. The randomisation list, the program that generated it and the random seed used will be stored in a secure network location, accessible only to those responsible for provision of the randomisation system. Clinicians responsible for delivering the perioperative care will perform the web-based randomisation.

Intervention

The choice of anaesthetic agents is influenced largely by the successful utilisation of these combinations in a previous study[8]; the ready availability of these drugs within the UK; acceptability to collaborating centres and the ability of the combination to provide both rapid onset and prolonged duration of block.[17]

For peer review only - http://bmjopen.bmj.com/site/about/guidelines.xhtml

Interventional arm: regional anaesthesia (RA)

An ultrasound guided supraclavicular or axillary BPB will be administered by a consultant anaesthetist trained in RA, or trainee practicing under direct supervision. The supraclavicular approach will be considered first-line, unless the anatomy or patient risk profile is unfavourable. In patients on antiplatelets or other anticoagulants, the choice between supraclavicular and axillary block will be at the anaesthetist's discretion, taking into account "compressibility, vascularity and consequences of bleeding".[18] Where pulmonary disease is present, an axillary nerve block eliminates the risk of pneumothorax or temporary phrenic nerve paralysis.

A 1:1 mixture of 0.5% L-bupivacaine and 1% lidocaine, mixed with epinephrine to 1 in 400,000 final concentration, will be utilised (Appendix 1). Maximum dose limits are 2 mg/kg for bupivacaine and 7 mg/kg for lidocaine with epinephrine, recognising the effects are additive. The volume of LA injected must account for patient weight and maximum dose limits, whilst considering the need for LA supplementation. In a study where the median patient weight was 66kg the ED_{95} for supraclavicular blocks was 27ml.[19] A minimum volume of 25ml must be injected for patients over 60kg. This is reduced to 20ml for patients weighing 51-60kg and 15ml in patients <45kg (Appendix 1). A suggested supraclavicular technique involves depositing a minimum of 25% of LA in the "corner pocket" between the 1st rib and the subclavian artery and the remainder posterolateral to the plexus, avoiding deliberate intracluster injection.[20] For axillary blocks, the same minimum volumes must be utilised, targeting 25% of the LA to the musculocutaneous nerve, with the remainder deposited around the ulnar, median and radial nerves as well as the cutaneous nerves of the arm and forearm if visualised.

For peer review only - http://bmjopen.bmj.com/site/about/guidelines.xhtml

Sensory and motor block of musculocutaneous, median, radial and ulnar nerves will be recorded every 5 minutes using a validated 3-point scale.[21] Sensory blockade of the medial cutaneous nerve of the forearm and arm will also be recorded. Measurements will be continued until either sensory block is adequate or 30-minutes has elapsed, at which point the block may be supplemented by targeted ultrasound-guided axillary or midhumeral injection as appropriate.

Comparator Arm: Local Anaesthesia (LA)

A 1:1 mixture of 0.5% L-bupivacaine and 1% lidocaine will be infiltrated around the operative site by the operating surgeon. After 5 minutes, adequacy of anaesthesia will be tested by application of a painful stimulus and additional LA infiltration administered as required. Maximum dose limits of 2 mg/kg for bupivacaine and 3mg/kg for lidocaine will be observed, recognising the effects are additive.

Management of a "failed block" (or failure of local anaesthesia)

A "failed block" will be defined as any block that despite the targeted intervention described above requires additional supplementation with LA, analgesia, conversion to GA or abandonment of surgery. The algorithm for "failed block" or "failed LA" will be as follows:

1. Supplementation at surgical site with LA (1% lidocaine) up to maximum cumulative LA dosage
2. Intravenous sedation and analgesia at the discretion of the anaesthetist
3. General anaesthesia
4. Abandonment of procedure: decision to be made following discussion between operating surgeon and anaesthetist if deemed unsafe to proceed with GA

For peer review only - http://bmjopen.bmj.com/site/about/guidelines.xhtml

The Trial Steering Committee (TSC)/Independent Data Monitoring Committee (IDMC) will monitor the number of failed blocks for patient safety and quality assurance throughout the study.

Fistula surgery

A standard approach to the vessels will be via a transverse incision at, or just below, the elbow crease for BCF and longitudinal or curvilinear incision at the wrist for RCF. The cephalic vein (or median cubital vein if suitable) will be dissected and skeletalised for a short length proximally and distally. Visible branches will ligated and divided. The vein will be divided, spatulated where appropriate and flushed with heparinised saline. The artery will be dissected and controlled with bulldog clamps or slings. The decision to utilise median cubital, perforating branch or true outflow cephalic vein for the anastomosis will be at the surgeon’s discretion, as will be the decision to create a proximal radial or ulnar-cephalic fistula at the elbow. The size of the arteriotomy will be based on individual patient risks and benefits but arteriotomies will generally be between 3-5mm in length on the brachial artery and 7-10mm on the radial artery. An end-to-side anastomosis of vein to artery will be performed with continuous 6.0 (elbow) or 7.0 (wrist) Prolene.

Blinding

Due to the systemic effects of RA (motor blockade; visible venodilatation etc.), which do not occur with LA, it will not be possible to blind the patients, surgical or anaesthetic teams. Dialysis staff, and staff performing follow-up visits will be blinded to the intervention. USS will also provide independent objective assessment of the AVF. The statisticians and health economist will be blinded to the intervention.

For peer review only

For peer review only - http://bmjopen.bmj.com/site/about/guidelines.xhtml

Outcomes

The primary outcome is unassisted functional AVF patency at one year, defined as the ability of the AVF to uninterruptedly deliver the prescribed dialysis without intervention.[22] In pre-dialysis patients, this will be assessed both clinically by an experienced, blinded dialysis nurse and ultrasonographically, the target being 4mm diameter and access flow >500ml/min.[23]

All secondary outcome measures will be assessed at 3 and 12 months. These were chosen with two considerations: patient-centred care and to facilitate health economic analysis. The secondary outcomes reflect the "standard CKD set" recommended by the International Consortium for Health Outcomes Measurement (ICHOM) CKD Working Group.[24] Key safety and efficacy outcomes for USS-guided regional nerve blocks outlined by the National Institute for Health and Clinical Excellence (NICE) will be recorded.[25] Secondary outcome measures are listed in Box 2. These include access specific outcomes e.g. re-interventions; patient specific outcomes e.g. mortality; HR-QOL outcomes e.g. KD-QOL; quality and speed of onset of anaesthesia; and safety outcomes.

Economic evaluation

A cost-effectiveness analysis will be conducted alongside the clinical trial. Two complementary cost-effectiveness analyses will be performed, namely a within-trial evaluation where cost and health effects of individual patients are limited to the one-year follow-up period of the trial and a decision model approach where effects are modelled to incorporate longer-term impacts of the intervention.

For peer review only - http://bmjopen.bmj.com/site/about/guidelines.xhtml

The primary outcome of the economic evaluation is the incremental cost-effectiveness ratio (ICER) of RA compared to LA in AVF creation expressed in cost per quality adjusted life year (£/QALY). All intervention resource use and access-related resource use will be derived from the secondary outcome measures and unit costs applied to all resource use estimates informed, where possible, from standard UK sources. A bottom-up approach will be used to estimate the costs associated with the two anaesthesia procedures. Effects will be captured at the individual patient level and QALYs will be derived by combining overall survival with utility weights derived from the EQ-5D questionnaire values obtained at the pre-operative time, at 3 months and 12 months after treatment.

A discrete-time state-transition Markov model will then be used to assess long-term economic impact of the intervention beyond the trial period, with each cycle consisting of relevant events (i.e. maturation/functional patency, failure, complications, re-intervention, alternative access, adverse events, death). Events will be driven by transition probabilities within the model, being informed partly by within-trial data in the short-term (i.e. up to one year) and other sources (literature, electronic health records, etc) in the long-term (i.e. beyond one year).

Retention/withdrawal criteria

Participants may voluntarily withdraw from the study at any time. However, due to the nature of the intervention, it is impossible to change the allocated treatment once the anaesthetic procedure has been performed. Follow-up visits will be timed to coincide with dialysis sessions to minimise follow-up burden and promote trial retention.

For peer review only

For peer review only - http://bmjopen.bmj.com/site/about/guidelines.xhtml

Data collection and data management

Study specific data, which is non-identifiable, will be collected on the electronic case report form (eCRF) using a unique patient identifier for reporting. Only the study site will have access to the identifiable information to maintain participant confidentiality. Pseudoanonymised data entered into the eCRF will be managed and stored by the RCB. The RCB systems are fully validated in accordance with industry and regulatory standards and incorporate controlled access security. Data integrity is assured by strictly controlled procedures, including secure data transfer procedures. A computer database will be constructed specifically for the trial data and will include range and logic checks to prevent erroneous data entry. Independent checking of data entry will be periodically undertaken on small sub-samples. The trial statistician will also regularly check the balance of allocations by stratification variables.

All essential documents will be archived in a secure commercial vault for a minimum of 5 years after completion of the trial. Trial data will be stored under controlled conditions for at least 10 years after closure. During this period, all data will be accessible to the competent authorities and the sponsor for audit and monitoring purposes with suitable notice.

Sample size

566 subjects (283 per arm) are required to detect a 15% difference in the primary outcome measure with 5% significance level and 90% power, assuming that 15% of subjects will be lost to follow-up, will change RRT modality, or die.

15% is considered to be the minimum clinically importance difference between the two cohorts. It is a conservative estimate of the 19% difference in one year unassisted functional

For peer review only - http://bmjopen.bmj.com/site/about/guidelines.xhtml

patency observed in the results from our single-centre RCT and is the magnitude of difference considered appropriate by experts following independent review of the protocol by the UK Renal Trials Network (UKRTN).[12] UK renal registry (UKRR) data indicates that 47% of incident patients currently commence HD via an AVF/AVG.[6] A 15% increase in functional patency would allow the Renal Association target of 60% to be achieved.

Statistical Analysis Plan

All statistical analyses will primarily be performed according to the intention-to-treat principle. However, additional analysis will be pre-specified to address "failed blocks" (e.g. per-protocol, as treated, and complier-average causal effects analyses). Baseline demographics will be summarised by treatment group without formal statistical comparison. The primary outcome will be analysed using logistic regression, adjusting for stratification variables used at randomisation and the treatment group assigned. The treatment effect will be reported with a 95% confidence interval for the Odds Ratio and p-value also reported. Time to loss of functional access will also be analysed using survival analysis regression methods. Similarly, for each of the secondary outcomes, analyses will be conducted using appropriate regression methods reporting the treatment effects, 95% confidence intervals and p-values. Safety data including the number of adverse events and serious adverse events will be reported overall and by study arm, where no formal statistical testing will be carried out.

Interim analysis and early termination criteria

A 4-month internal pilot will be employed, principally to assess feasibility of recruitment. Stop-go (traffic light) criteria for continuance to the full trial will be used:

For peer review only - http://bmjopen.bmj.com/site/about/guidelines.xhtml

- Red: Stop if <50 patients recruited or if <5 centres are open to recruitment
- Amber: Enrol more centres if between 48-95 patients recruited
- Green: Continue within existing parameters if >95 patients recruited

If there is failure of adherence to trial protocol in >20% of participants or significant safety concerns are raised by the IDMC the trial will not progress beyond the pilot phase. In the event that the trial was to be terminated following the internal pilot, all patients would be followed up until the end of trial.

Embedded process evaluation study

An embedded process evaluation study will run in parallel with the trial. The rapid feedback evaluation approach delivered by the Rapid Research Evaluation and Appraisal Lab (RREAL) at the Department of Targeted Intervention, University College London (UCL) will combine qualitative data obtained from semi-structured interviews with patients, carers and staff and documentary analysis (reports, meeting minutes etc.) to:

- Explore staff views and experiences with different approaches to recruitment
- Examine patient and carer experiences in trial participation (understanding of trial literature; experience with treatment options; reasons for withdrawal)
- Examine patient and carer experiences of declining to take part in the trial
- Identify barriers and enablers to trial set-up, recruitment and delivery from the point of view of staff.

Data obtained will be analysed and shared with researchers throughout the main ACCess study at a time when they can be used to inform within trial decision-making processes.[26]

Adverse event reporting

For peer review only - http://bmjopen.bmj.com/site/about/guidelines.xhtml

In accordance with the Research Governance Framework for Health and Community Care, any untoward medical occurrence in a trial participant will be considered an adverse event (AE), recorded in the patient's case notes and assessed for severity.[27] Any adverse event that is life-threatening; results in death, birth defect or significant disability; or requires hospitalisation is considered a Serious Adverse Event (SAE). The following trial-specific adverse events will also be considered SAEs:

- A recognised perioperative complication of regional or local anaesthetic administration (including pneumothorax, inadvertent arterial puncture, inadvertent intraneural/intravascular injection, persistent neuropraxia, LA toxicity)
- The requirement for re-exploration or abandonment of surgery

Full details including the nature of the event, start and stop dates, severity, actions taken, relationship to the trial specific intervention and outcome of all SAEs will be reported to the sponsor, via the Glasgow Clinical Trials Unit, on the eCRF and events followed-up until satisfactory resolution. All SAE will be assessed for causality and expectedness. Any SAE believed to be related to a trial specific procedure that is thought to be unexpected (i.e. the event is not listed within the protocol nor would not be expected to occur when carrying out the trial specific procedure in normal clinical practice) will be considered a Related Unexpected Serious Adverse Event (RUSAE) and must be reported to the sponsor within 24 hours of the site becoming aware. All SAEs will be reported to the IDMC, TSC and sponsor. The sponsor must inform the REC of any RUSAE within 15 days.

Trial management and audit

The TSC, including a patient representative, will provide overall supervision of the trial and ensure that trial conduct is in line with standards set out in the EU Good Clinical Practice

For peer review only

(GCP) Guideline.[28] The TSC (including the Chief Investigator, trial statistician and five independent experts) will meet on six occasions during the trial, review blinded safety data biannually and report formally to the sponsor.

An IDMC will be responsible for monitoring data emerging from the trial, in particular as they relate to the safety of participants. The IDMC will be completely independent of the trial and any institutions involved in the trial. It will consist of an expert clinical trialist (chair); expert in the field of vascular access and expert statistician. The IDMC will meet annually during the recruitment and follow-up phases of the trial. The IDMC is the only body that will have access to the unblinded comparative data during the trial. Ultimate responsibility in deciding whether or not to act upon recommendations from the IDMC or a decision for early termination lies with the TSC in conjunction with the sponsor and funder.

Following risk assessment, it has been determined that the study will not be routinely monitored by the sponsor, however the sponsor randomly selects a number of studies to be audited annually. In addition audits can be requested by individual participating sites/TSC.

Public and patient involvement

Patients and the public have been integral to the design and implementation of this trial. Consultation and focus groups identified both the importance of access functionality, which is reflected in our choice of unassisted functional patency as the primary outcome measure, and the "exhaustion and loss of control" experienced by patients on dialysis. The trial protocol reflects these challenges such that follow-up will, where possible, be

For peer review only - http://bmjopen.bmj.com/site/about/guidelines.xhtml

performed on whilst the patient is on dialysis to minimise the burden additional unnecessary hospital visits and cannulation diaries have been developed so that patients participating in the study will have some ownership for collecting data and reporting outcomes. The embedded process evaluation study will also explore patient and carer experiences of trial participation. A patient representative will sit on the TSC to ensure that the patient's voice is heard throughout the trial. Study participants will receive results via their dialysis units, social media, renal charities and patient groups.

Ethics and dissemination

The trial protocol has been approved by the West of Scotland Research and Ethics Committee (REC) 3 (20/WS/0178). Research will be conducted in accordance with the Declaration of Helsinki, the Principles of Good Clinical Practice, the Data Protection Act (2018), the General Data Protection Regulation and the UK Policy Framework for Health and Social Care Research. Substantial amendments that require review by REC will not be implemented until the REC grants a favourable opinion for the trial (amendments may also need to be reviewed and approved by the NHS R&D departments before they can be implemented in practice at local sites).

Consent

A member of the research team will obtain written informed consent prior to administration of any trial intervention (Appendix 2). Participants will also be asked to provide consent for future data-linkage studies via the Scottish Renal Registry (SRR) and UK Renal Registry (UKRR). All patients will have the right to refuse participation and withdraw

For peer review only

from the trial at any time without providing reasons and without prejudicing further treatment.

Access to data

The anonymised participant level dataset and statistical code of generating the results will be made publicly available within 12 months of the end of trial via an online data repository.

Ancillary and post-trial care

The anaesthetic (both regional and local) reflects a single event intervention, therefore contingency plans for the provision of ongoing treatment for individual trial participants is not required. The sponsor is a member of the Clinical Negligence and Other Risks Indemnity Scheme (CNORIS), which covers the Sponsors legal liability in relation to clinical trials including clinical negligence and harm from study design.

Publication/Dissemination

Ownership of the data arising from this study resides with the grant holders. Research findings will be published in the name of the ACCess Collaborative Group, acknowledging the writing group as authors. Results of this trial will have implications for patients and clinicians across a range of disciplines including nephrology, anaesthesia, vascular access surgery, and dialysis nursing. The principal target audience however is healthcare commissioners and policy makers. Results will be published in open access peer-reviewed journals within 12 months of completion of the trial. We will also present our findings key national/international renal meetings and support dissemination of trial outcomes directly to patients via patient groups, renal charities and social media.

For peer review only

Collaborators

Gavin Pettigrew, Regin Lagaac (Addenbrooke's Hospital, Cambridge University Hospitals NHS Foundation Trust), Rosie Hogg (Belfast City Hospital, Belfast Health and Social Care Trust), Stuart Suttie, Andrew Dalton, Samira Bell, Rose Ross (Ninewells Hospital, NHS Tayside), Thalakunte Muniraju, David McNair, Linda Stiff, Catherine Jardine (Dumfries and Galloway Royal Infirmary, NHS Dumfries & Galloway), Rita Singh, Mohammed Tariq Dosani, Jennifer Sainsbury (The Freeman Hospital, Newcastle-upon-Tyne Hospitals NHS Foundation Trust), Nikolaos Karydis, Kiran Sran, Kariem El-Boghdadly, Nadia Castrillo (Guy's and St Thomas' NHS Foundation Trust, London), James Gilbert, Sanjay Sinha, Sheera Sutherland, Sarah Crosbie, Madita Gavrila (Churchill Hospital, Oxford University Hospitals NHS Foundation Trust), Alex Vesey, Sandra Montgomery, Tina McLennan, Nina Tarkowska, Scott Oliver, Liz Brown, Shelley McLachlan (Monklands and Hairmyres Hospitals, NHS Lanarkshire), Jonathan de Siqueira, Max Troxler, Nikki Dewhirst, Mark Wright, Chetan Srinath (Leeds General Infirmary, Leeds Teaching Hospitals NHS Trust), Philip Bennett, Darren Morrow (Norfolk and Norwich University Hospitals NHS Foundation Trust Norwich), Emma Aitken, Marc Clancy, Iain Thomson, Andrew Jackson, Karen Stevenson, David Kingsmore, Margaret Aitken (Queen Elizabeth University Hospital, Glasgow, NHS Greater Glasgow and Clyde), Reza Motallebzadeh, Vishal Nangalia, Vashist Deelchand, Rani Badhan (Royal Free London NHS Trust, London), Rajesh Sivaprakasam, Gareth Ackland Tim Egan, Matt Wikner (The Royal London Hospital, Barts Health NHS Trust), Alan Macfarlane, Rachel Kearns (Stobhill Ambulatory Care Hospital, Glasgow, NHS Greater Glasgow and Clyde), Lucian Gaianu (Independent Health Economist), Alex MacConnachie, Kirsty Wetherall,

For peer review only

Patrick Mark (University of Glasgow), Ramani Moonsinghe, Cecilia Vindrola (University College London).

Authors' contributions

AJRM, RJK, MC, DBK, KS, AJ, PBM, MA, RM, CV, LG, GJP, RM, NK, AV, RS, TM, SS, AMcC, KW, KE, RH, IT, VN, EA contributed to the development and implementation of this protocol and have approved the manuscript.

Funding

This work is supported by National Institute for Health Research (NIHR) [Health Technology Assessment (HTA) 130567]. The views expressed are those of the authors and not necessarily those of the NIHR or the Department of Health and Social Care.

Competing interests

AJRM is currently President of Regional Anaesthesia UK (RA-UK). RA-UK has reviewed and endorsed the trial protocol, but has not been involved in the design or development in any way. AJRM has received consulting fees from Heron therapeutics and Intelligent US. RH has received honoraria from GE.

Sponsor

The trial sponsor is NHS Greater Glasgow and Clyde

Sponsor's Representative

For peer review only

Dr Maureen Travers, Senior R&D Co-ordinator, NHS Greater Glasgow and Clyde Email:

Maureen.Travers@ggc.scot.nhs.uk

Project Manager

Lisa Jolly, R&D Project Manager, NHS Greater Glasgow and Clyde. Email:

Lisa.Jolly@ggc.scot.nhs.uk

Patient consent for publication

Not required

Acknowledgements

The authors would like to acknowledge the contribution of Ewen Maclean, patient and public involvement representative for trial design and implementation and Chloe Knott, patient representative on the TSC.

Twitter

@study_access

For peer review only

References

1. Scottish Renal Registry (2018) Annual Report. Available online at: https://www.srr.scot.nhs.uk/ Publications/docs/2018-10-09-SRR-Report.pdf?4.
2. USRDS (2014). USRDS Annual Data Report 2014: Volume 2, End stage renal disease in the United States. https://www.usrds.org/2013/pdf/v2_esrd_full_13.pdf.
3. National Kidney Care Healthcare Quality Improvement Partnership (2011) National Kidney Care Vascular Access Audit Report 2011
4. Wasse H, Kutner N, Zhang R & Huang Y. Association of Initial Haemodialysis Vascular Access with Patient-Reported Health Status and Quality of Life. *Clin J Am Soc Nephrol* 2007*;* **2**: 708-14.
5. Malas MB, Canner JK, Hicks CW et al.Trends in incident vascular access and mortality. *JAMA Surgery* 2015; **150**: 441-8.
6. UK Renal Registry (2017) 20th Annual Report. https://www.renalreg.org/reports/2017-twentieth-annual-report
7. Kumwenda M, Mitra S & Reid C (2017) Clinical Practice Guideline: Vascular Access for Haemodialysis https://renal.org/sites/renal.org/files/vascular-access.pdf
8. Aitken E, Jackson A, Kearns R et al. Effect of regional vs local anaesthesia on outcome after arteriovenous fistula creation: randomised controlled trial. *Lancet* 2016; **388**:1067-74.
9. Dember L, Beck G, Allon M et al. Effect of clopidogrel on early failure of arteriovenous fistulas for hemodialysis: randomised controlled trial. *JAMA* 2008*;* **299**: 2164-71.
10. Keuler J, Weiss C, Klemm K et al. Assessing changes in tissue oxygenation by near infrared spectroscopy following brachial plexus block for arteriovenous fistula surgery: A prospective observational pilot study. *Eur J Anaesthesiol* 2018*;* **35**: 759-65.
11. Cerneviciute R, Sahebally M, Ahmed K et al. Regional versus local anaesthesia for haemodialysis arteriovenous fistula formation: systematic review and meta-analysis *Eur J Vasc Endovasc Surg* 2017; **53**: 34-42.
12. Aitken E, Kearns R, Gaianu L, et al. Long-term functional patency and cost-effectiveness of arteriovenous fistula creation under regional anaesthesia: a randomised controlled trial *J Am Soc Nephrol* 2020*;* **31**: 1871-82.
13. Schmidli J , Widmer MW, Basile C et al. Vascular Access: 2018 Clinical Practice Guidelines of the European Society for Vascular Surgery*. Eur J Vasc Endovasc Surg* 2018; **55**: e757-e818.
14. ERA-EDTA. Clinical Practice Guideline on peri- and post-operative care of arteriovenous fistulas and grafts for haemodialysis in adults. http://www.european-renal-best-practice.org/sites/defailut/files/u33/ERBP_006-VASCAC.ShortVersion.pdf.
15. Lok CE, Huber TS, Lee T et al. KDOQI Clinical Practice Guidelines for Vascular Access:2019 update. *Am J Kid Dis* 2020; **75**: S1-164.

For peer review only - http://bmjopen.bmj.com/site/about/guidelines.xhtml

16. Armstrong RA, Wilson C, Elliot L et al. Regional anaesthesia for arteriovenous fistula formation surgery. *Anaesthesia* 2020; **75**: 626-33.
17. Bradley T, Teare T & Milner Q. Anaesthetic management of patients requiring vascular access surgery for renal dialysis. *BJA Education* 2017*;* **17**: 269-74.
18. Horlocker TT, Vandermeulen E, Kopp SI et al. Regional anaesthesia in the patient receiving antithrombotic or thrombolytic therapy *Reg Anes Pain Med* 2018; **43**: 263-309.
19. Kant A, Gupta PK, Zohar S, Chevret S, Hopkins PM. Application of the continual reassessment method to dose-finding studies in regional anesthesia: an assessment of the ED95 dose for 0.5 bupivacaine for ultrasound-guided supraclavicular block. *Anesthesiology* 2013; **119**: 29-35.
20. Gadsden J and Orebaugh S. Targeted intracluster supraclavicular brachial plexus block: too close for comfort. *Br J Anaesth* 2019*;* **122**: 713-5.
21. Rodriguez J, Taboada-Muniz M, Barcena M, Alvarez J Median versus musculocutaneous nerve response with single-injection infraclavicular coracoid block. *Reg Anesth Pain Med* 2014*;* **29**: 534–8.
22. Beathard GA, Lok CE, Glickman MH et al. Definitions and End Points for Interventional Studies for Arteriovenous Dialysis Access. *Clin J Am Soc Nephrol* 2018; **13**: 501-12.
23. Robbins M, Chamberlain N, Lockhart M et al. Hemodialysis arteriovenous fistula maturity: US evaluation. *Radiology* 2002; **225**: 59-64.
24. Verberne WR, Das-Gupta Z, Allegretti A et al. Development of an International Standard Set of Value-Based Outcome Measures for Patients with Chronic Kidney Disease: A report of the ICHOM CKD Working Group. *Am J Kidney Dis* 2019*;* **73**: 372-84.
25. NICE. Interventional procedure overview of ultrasound-guided regional nerve block. Available online at: https://www.nice.org.uk/guidance/IPG285
26. McNall M, Welch V, Ruh K et al. (2004) The use of rapid-feedback evaluation methods to improve the retention rates of an HIV/AIDS healthcare intervention. *Evaluation and Program Planning;* 27: 287-294.
27. UK policy framework for health and social care research. NHS Health research Authority 2020. https://s3.eu-west-2.amazonaws.com/www.hra.nhs.uk/media/documents/Final_Accessibility_uk-policy-framework-health-social-care-research_.pdf
28. International Conference on Harmonization (ICH) guideline on Good Clinical Practice (1996) http://www.ich.org/products/guidelines/efficacy/efficacy-single/article/good-clinical-practice.html
29. Brimble KS, Rabbat C, Treleaven DJ, Ingram AJ. Utility of ultrasonographic venous assessment prior to forearm arteriovenous fistula creation. *Clin Nephrol* 2002*;* **58**: 122-7.
30. AAGBI, Obstetric Anaesthetists Association and RA-UK (2013) Regional anaesthesia and patients with abnormalities of coagulation. *Anaesthesia* 2013*;* **68**: 966-72.
31. ED-5D User Guide, EuroQOL Group. Available online at: https://euroqol.org/wp-content/uploads/2016/09/EQ-5D-5L_UserGuide_2015.pdf

For peer review only

32. Kidney Disease Quality of Life Short Form (KDQOL-SF). The Rand Corporation. Available online at: https://www.rand.org/health-care/surveys_tools/kdqol.html
33. Richarz S, Greenwood S, Kingsmore DB, Thomson P, Dunlop M, Bouamrane M, Meiklem R, Stevenson K (2021) Validation of a Vascular Access Specific Quality of Life Measure (VASQoL), *Journal of Vascular Access,* in press

For peer review only

Box 1. Inclusion and exclusion criteria.

Inclusion criteria

- All adult patients (≥18 years old) with kidney failure requiring KRT or CKD stage IV or V referred for primary RCF or BCF creation

Exclusion criteria

General

- Patients unable or unwilling to provide informed consent
- Patient preference for general or alternative anaesthesia
- Active infection at surgical or anaesthetic site

Access specific

- Previous ipsilateral AVF creation (a previous attempt at distal AVF creation which fails immediately is not considered a contraindication, however any distal access which has previously run sufficiently to mature the outflow vein or proximal revision of an existing AVF is considered a contraindication)
- Known ipsilateral cephalic arch or central venous stenosis (even if previously treated)
- USS evidence of stenosis in inflow artery
- Radial or brachial artery <1.8mm diameter and/or cephalic vein <2mm at wrist or <3mm at elbow (with tourniquet) on pre-operative USS[29]

Contraindications to anaesthetic agents/ technique:

- Allergy to LA or any excipient agents
- Acquired or inherited coagulopathy (including warfarin/ heparin/ novel oral anticoagulant use where it has not been possible to stop the anticoagulation in anticipation of surgery) and/or platelets <75 or INR > 1.4[30]
- Significant pre-existing neurological disorder affecting upper limb
- Weight <45kg

For peer review only

Box 2: Outcome measures

Primary outcome measure

Unassisted functional patency of the index fistula at one-year

Secondary outcome measures

Access specific outcome measures

- Patency (i.e. is the fistula running ?): defined clinically as the presence of a bruit
- Access complications (including infection, stenosis, thrombosis, steal, bleeding)
- Re-operation/re-intervention to maintain or re-establish patency (revisional surgery, angioplasty, stenting or thrombectomy)
- Alternative accesses e.g. CVCs
- Time to first cannulation
- Cannulation difficulties (including failure to establish two needle dialysis, infiltration, prolonged bleeding)

Patient specific outcome measures:

- Mortality
- Date commenced on HD
- Access modality at start of HD
- Change of RRT modality
- Change of access modality
- Access-related hospitalization

HR-QoL:

- EQ-5D-5L (EuroQol)[31] (crude health status measure; cost-effectiveness analysis)
- Kidney Disease Quality of Life Short Form (KDQOL-SF)[32] (renal specific HR-QOL)
- Vascular Access Specific Quality Of Life (VASQOL)[33] (vascular access specific HR-QOL)

Anaesthesia:

- Pain score at incision, at 30 minutes and 1 hour postoperatively (NRS 0-10)
- Speed of onset/quality of motor and sensory block[21]
- Need for anaesthetic supplementation
- ”Failed block”
- Volume of anaesthetic agent (mL)

For peer review only - http://bmjopen.bmj.com/site/about/guidelines.xhtml

- Time to administer anaesthetic (mins)

Other:

- Change in surgical plan e.g. switch from BCF to RCF prior to surgical incision

Figure 1. Participant timeline.

Appendix 1. Preparation of anaesthetic mixture for RA and administration of brachial plexus block.

Appendix 2. Consent form.

For peer review only

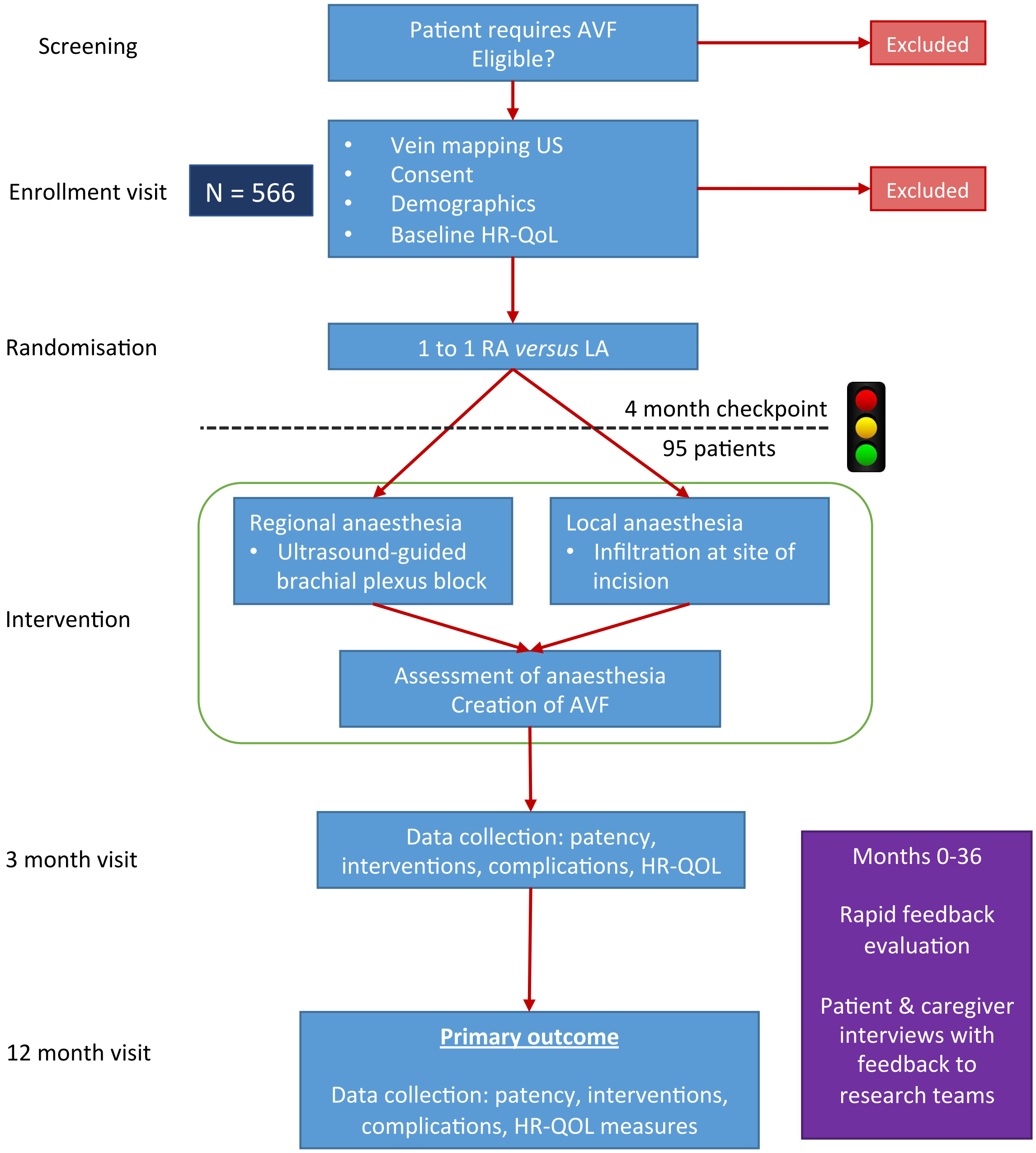
Screening
Patient requires AVF
Eligible?
Excluded
Enrollment visit
N = 566
Vein mapping US
Consent
Demographics
Baseline HR-QoL
Excluded
Randomisation
1 to 1 RA versus LA
4 month checkpoint
95 patients
Intervention
Regional anaesthesia
Ultrasound-guided brachial plexus block
Local anaesthesia
Infiltration at site of incision
Assessment of anaesthesia
Creation of AVF
3 month visit
Data collection: patency, interventions, complications, HR-QOL
Months 0-36
Rapid feedback evaluation
Patient & caregiver interviews with feedback to research teams
12 month visit
Primary outcome
Data collection: patency, interventions, complications, HR-QOL measures

Appendix 1. Preparation of anaesthetic mixture for RA and administration of brachial plexus block.

Preparation:
Prepare 2 x 20ml syringes.
To each syringe add:

- 10ml 0.5% Levobupivacaine
- 10ml 1% Lidocaine
- 0.05ml 1:1,000 Adrenaline

Administration of brachial plexus block:
A minimum volume based on weight (outlined below) must be injected during the initial block (whether supraclavicular or axillary).

- 45-50kg: 15ml
- 51-60kg: 20ml
- >60kg: 25ml

Larger volumes may be used at the discretion of the anaesthetist as long as maximum dose limits are observed, remembering that local anaesthetic may also be required for surgical supplementation and that these doses are additive. Consider using ideal body weight in obese patients.

For peer review only

ACCESS

Anaesthesia Choice for Creation of ArtEriovenous FiStulae (ACCess study)

Chief Investigator: Emma Aitken

Participant Identification Number:

Please initial each box if you agree with the following statements:

I confirm that I have read and understood the information sheet dated *XX/XX/XX* (version *X.X)* for the above study. I have had the opportunity to consider the information, ask questions and have had those questions answered satisfactorily.	
I understand that my participation is voluntary and that I am free to withdraw at any time without giving any reason, without my medical care or legal rights being affected.	
I understand that relevant sections of my medical notes and data collected during the study may be looked at by individuals from the research team, regulatory authorities or from the Sponsor, NHS Greater Glasgow and Clyde, where it is relevant to my taking part in the research. I give permission to these individuals to have access to my records. I understand that my data will be held by the University of Glasgow.	
I understand that the information collected about me will be used to support other research in the future, and may be shared anonymously with other researchers.	
I agree to the study team having my phone number for the purpose of contacting me during the study.	
I agree to take part in the above study.	

Name of Participant **Signature** **Date**

....

Name of Researcher **Signature** **Date**

....

When completed: 1 for participant; 1 for researcher site file; 1 to be kept in medical notes.

For peer review only - http://bmjopen.bmj.com/site/about/guidelines.xhtml

SPIRIT 2013 Checklist: Recommended items to address in a clinical trial protocol and related documents*

Section/item	Item No	Description
**Administrative information**		
Title	1 Y	Descriptive title identifying the study design, population, interventions, and, if applicable, trial acronym
Trial registration	2a Y	Trial identifier and registry name. If not yet registered, name of intended registry
	2b Y	All items from the World Health Organization Trial Registration Data Set
Protocol version	3 Y	Date and version identifier
Funding	4 Y	Sources and types of financial, material, and other support
Roles and responsibilities	5a Y	Names, affiliations, and roles of protocol contributors
	5b Y	Name and contact information for the trial sponsor
	5c Y	Role of study sponsor and funders, if any, in study design; collection, management, analysis, and interpretation of data; writing of the report; and the decision to submit the report for publication, including whether they will have ultimate authority over any of these activities
	5d Y	Composition, roles, and responsibilities of the coordinating centre, steering committee, endpoint adjudication committee, data management team, and other individuals or groups overseeing the trial, if applicable (see Item 21a for data monitoring committee)
**Introduction**		
Background and rationale	6a Y	Description of research question and justification for undertaking the trial, including summary of relevant studies (published and unpublished) examining benefits and harms for each intervention
	6b Y	Explanation for choice of comparators
Objectives	7 Y	Specific objectives or hypotheses
Trial design	8 Y	Description of trial design including type of trial (eg, parallel group, crossover, factorial, single group), allocation ratio, and framework (eg, superiority, equivalence, noninferiority, exploratory)
**Methods: Participants, interventions, and outcomes**		
Study setting	9 Y	Description of study settings (eg, community clinic, academic hospital) and list of countries where data will be collected. Reference to where list of study sites can be obtained

For peer review only

Eligibility criteria	10 Y	Inclusion and exclusion criteria for participants. If applicable, eligibility criteria for study centres and individuals who will perform the interventions (eg, surgeons, psychotherapists)
Interventions	11aY	Interventions for each group with sufficient detail to allow replication, including how and when they will be administered
	11bY	Criteria for discontinuing or modifying allocated interventions for a given trial participant (eg, drug dose change in response to harms, participant request, or improving/worsening disease)
	11cY	Strategies to improve adherence to intervention protocols, and any procedures for monitoring adherence (eg, drug tablet return, laboratory tests)
	11dY	Relevant concomitant care and interventions that are permitted or prohibited during the trial
Outcomes	12 Y	Primary, secondary, and other outcomes, including the specific measurement variable (eg, systolic blood pressure), analysis metric (eg, change from baseline, final value, time to event), method of aggregation (eg, median, proportion), and time point for each outcome. Explanation of the clinical relevance of chosen efficacy and harm outcomes is strongly recommended
Participant timeline	13 Y	Time schedule of enrolment, interventions (including any run-ins and washouts), assessments, and visits for participants. A schematic diagram is highly recommended (see Figure)
Sample size	14 Y	Estimated number of participants needed to achieve study objectives and how it was determined, including clinical and statistical assumptions supporting any sample size calculations
Recruitment	15 Y	Strategies for achieving adequate participant enrolment to reach target sample size
**Methods: Assignment of interventions (for controlled trials)**		
Allocation:		
Sequence generation	16aY	Method of generating the allocation sequence (eg, computer-generated random numbers), and list of any factors for stratification. To reduce predictability of a random sequence, details of any planned restriction (eg, blocking) should be provided in a separate document that is unavailable to those who enrol participants or assign interventions
Allocation concealment mechanism	16bY	Mechanism of implementing the allocation sequence (eg, central telephone; sequentially numbered, opaque, sealed envelopes), describing any steps to conceal the sequence until interventions are assigned
Implementatio n	16cY	Who will generate the allocation sequence, who will enrol participants, and who will assign participants to interventions
Blinding (masking)	17aY	Who will be blinded after assignment to interventions (eg, trial participants, care providers, outcome assessors, data analysts), and how

	17bY	If blinded, circumstances under which unblinding is permissible, and procedure for revealing a participant's allocated intervention during the trial
**Methods: Data collection, management, and analysis**		
Data collection methods	18aY	Plans for assessment and collection of outcome, baseline, and other trial data, including any related processes to promote data quality (eg, duplicate measurements, training of assessors) and a description of study instruments (eg, questionnaires, laboratory tests) along with their reliability and validity, if known. Reference to where data collection forms can be found, if not in the protocol
	18bY	Plans to promote participant retention and complete follow-up, including list of any outcome data to be collected for participants who discontinue or deviate from intervention protocols
Data management	19 Y	Plans for data entry, coding, security, and storage, including any related processes to promote data quality (eg, double data entry; range checks for data values). Reference to where details of data management procedures can be found, if not in the protocol
Statistical methods	20aY	Statistical methods for analysing primary and secondary outcomes. Reference to where other details of the statistical analysis plan can be found, if not in the protocol
	20bY	Methods for any additional analyses (eg, subgroup and adjusted analyses)
	20cY	Definition of analysis population relating to protocol non-adherence (eg, as randomised analysis), and any statistical methods to handle missing data (eg, multiple imputation)
**Methods: Monitoring**		
Data monitoring	21aY	Composition of data monitoring committee (DMC); summary of its role and reporting structure; statement of whether it is independent from the sponsor and competing interests; and reference to where further details about its charter can be found, if not in the protocol. Alternatively, an explanation of why a DMC is not needed
	21bY	Description of any interim analyses and stopping guidelines, including who will have access to these interim results and make the final decision to terminate the trial
Harms	22Y	Plans for collecting, assessing, reporting, and managing solicited and spontaneously reported adverse events and other unintended effects of trial interventions or trial conduct
Auditing	23Y	Frequency and procedures for auditing trial conduct, if any, and whether the process will be independent from investigators and the sponsor
**Ethics and dissemination**		
Research ethics approval	24Y	Plans for seeking research ethics committee/institutional review board (REC/IRB) approval

Protocol amendments	25Y	Plans for communicating important protocol modifications (eg, changes to eligibility criteria, outcomes, analyses) to relevant parties (eg, investigators, REC/IRBs, trial participants, trial registries, journals, regulators)
Consent or assent	26aY	Who will obtain informed consent or assent from potential trial participants or authorised surrogates, and how (see Item 32)
	26bY	Additional consent provisions for collection and use of participant data and biological specimens in ancillary studies, if applicable
Confidentiality	27Y	How personal information about potential and enrolled participants will be collected, shared, and maintained in order to protect confidentiality before, during, and after the trial
Declaration of interests	28Y	Financial and other competing interests for principal investigators for the overall trial and each study site
Access to data	29Y	Statement of who will have access to the final trial dataset, and disclosure of contractual agreements that limit such access for investigators
Ancillary and post-trial care	30Y	Provisions, if any, for ancillary and post-trial care, and for compensation to those who suffer harm from trial participation
Dissemination policy	31aY	Plans for investigators and sponsor to communicate trial results to participants, healthcare professionals, the public, and other relevant groups (eg, via publication, reporting in results databases, or other data sharing arrangements), including any publication restrictions
	31bY	Authorship eligibility guidelines and any intended use of professional writers
	31cY	Plans, if any, for granting public access to the full protocol, participant-level dataset, and statistical code
**Appendices**		
Informed consent materials	32Y	Model consent form and other related documentation given to participants and authorised surrogates
Biological specimens	33 N/A	Plans for collection, laboratory evaluation, and storage of biological specimens for genetic or molecular analysis in the current trial and for future use in ancillary studies, if applicable

*It is strongly recommended that this checklist be read in conjunction with the SPIRIT 2013 Explanation & Elaboration for important clarification on the items. Amendments to the protocol should be tracked and dated. The SPIRIT checklist is copyrighted by the SPIRIT Group under the Creative Commons "Attribution-NonCommercial-NoDerivs 3.0 Unported" license.

For peer review only
